# Orphan Medicine Incentives: How to Address the Unmet Needs of Rare Disease Patients by Optimizing the European Orphan Medicinal Product Landscape Guiding Principles and Policy Proposals by the European Expert Group for Orphan Drug Incentives (OD Expert Group)

**DOI:** 10.3389/fphar.2021.744532

**Published:** 2021-12-16

**Authors:** Annemieke Aartsma-Rus, Marc Dooms, Yann Le Cam

**Affiliations:** ^1^ Department of Human Genetics, Leiden University Medical Center, Leiden, Netherlands; ^2^ University Hospitals Leuven, Leuven, Belgium; ^3^ EURORDIS, Brussels, Belgium; ^4^ The OD Expert Group, Brussels, Belgium; ^5^ Copenhagen Economics A/S, Copenhagen, Denmark

**Keywords:** orphan medicine, orphan drug, rare disease, incentives, unmet need, OMP regulation

## Abstract

Today policy makers face the challenge to devise a policy framework that improves orphan medicinal product (OMP) development by creating incentives to deliver treatments where there are none and to authorize innovative and transformative treatments where treatments already exist. The European Expert Group on Orphan Drug Incentives (hereafter, OD Expert Group) came together in 2020 to develop policy proposals to facilitate EU policy makers to meet this challenge. The group brings together representatives of the broad rare disease community, including researchers, academia, patient representatives, members of the investor community, rare disease companies and trade associations. The group’s work builds on the recognition that only an ambitious policy agenda developed in a multi-stakeholder setting can bring about the quantum leap needed to address unmet needs of rare disease patients today. Along the OMP development path, the OD Expert Group has identified four main needs that a policy revision should address: 1) Need to improve the R&D ecosystem for basic research and company take-up of development. 2) Need to improve the system of financial incentives and rewards. 3) Need to improve the flexibility, predictability and speed of the regulatory pathway. 4) Need to improve the coherence and predictability of demand and pricing for OMPs. This article presents the results of the OD Expert Group work as a set of guiding principles that the revision of the policy framework should follow and a set of 14 policy proposals that address the main needs of OMP development in Europe today.

## Introduction

Rare diseases are diseases with a particularly low prevalence. In the European Union 2000 (EU), a disease is considered rare when it affects less than 5 per 10,000 people (European Commission 2020a, 5).

While the number of persons suffering from an individual rare disease is small, overall, rare diseases affect many Europeans. Currently, we know of over 6,000 rare diseases affecting approximately 30 million Europeans, i.e., 6% of the European population (Wakap et al., 2020). In addition, 80% of rare diseases are of genetic origin and are chronic and life-threatening. For most rare diseases there is no authorised treatment available (Tambuyzer et al., 2020).

In and by itself, the process for developing and bringing medicines to the market is complex, costly, and requires the collaboration of many stakeholders (researchers, industry, patients, medical professionals, investors, funding bodies and regulators).

While any medicinal development path is costly and failure-ridden, the complexities are even higher for orphan medicinal products (OMPs). The small number of patients affected by a given rare disease may mean that it attracts relatively less attention and funding in the research community, makes research and clinical trial studies more difficult and riskier, makes regulatory approval more difficult to achieve and, overall, makes the investment case less attractive for OMP developers. For example, small clinical trials means it is more risky to predict the effect on a larger number of patients outside of the inclusion criteria. As real world efficacy is difficult to predict regulatory approval and marketing are more challenging.

**FIGURE 1:**
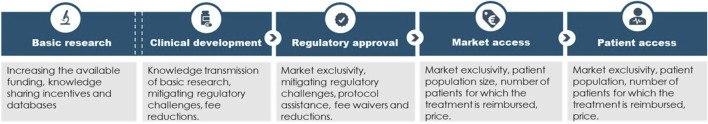
Incentives for OMP development. Source: The OD Expert Group

Given these features, incentivising the development of medicinal products to address rare diseases *OMPs*) is not an easy task. We define an incentive in this context as any measure meant to promote the development of medicines to treat rare diseases (European Commission 2020a). Various types of incentives are available to policy makers to increase research in and the development of OMPs, see Figure 1.

Against that background, the EU OMP Regulation, introduced in 2000, aimed at ensuring higher availability of OMPs through a specific set of incentives (European Commission 1999, European Commission 2000): a 10-year market exclusivity period for designated OMPs, protocol assistance from the European Medicines Agency (EMA), fee reductions during the approval process, and EU-funded research for OMP development aimed at increasing research in rare diseases. The OMP Regulation also invited Member States to provide national incentives, such as tax benefits.

Next to the OMP Regulation, the wider regulatory landscape, including for instance the EU Clinical Trials Directive (European Commission 2001) and national pricing and reimbursement procedures, influences development incentives for OMPs.

**FIGURE 2:**
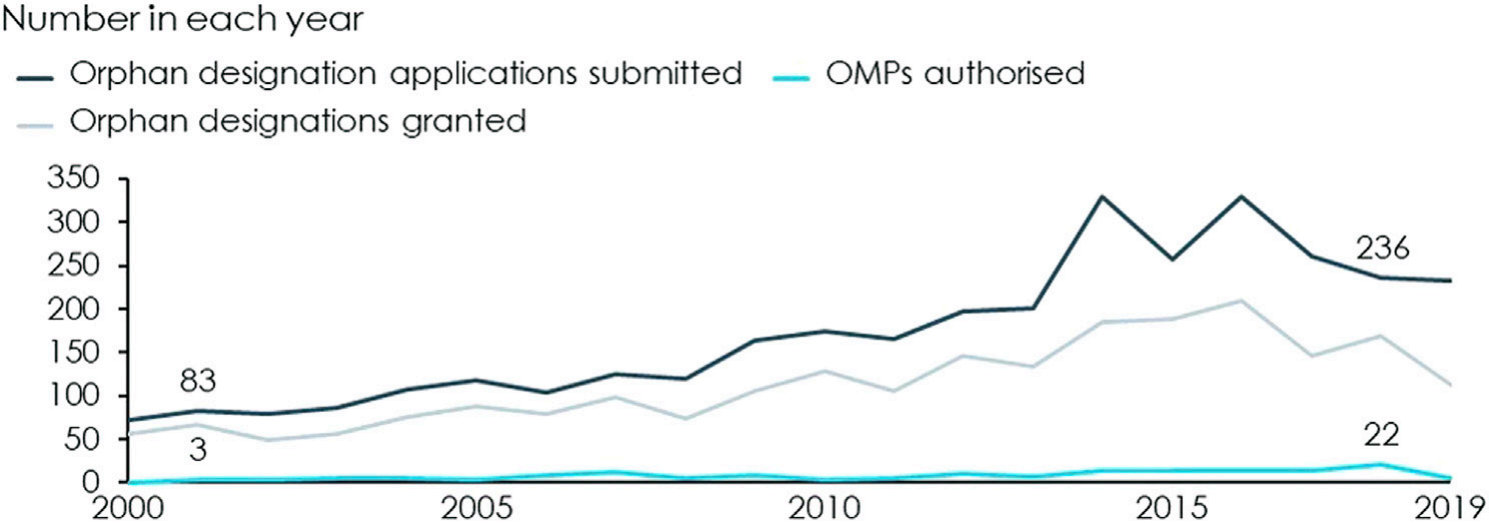
Applications submitted, designations granted and authorised OMPs by year. Source: European Commission (2020a) and European Medicines Agency (2020). These also contain applications and OMP that have been withdrawn.

The advent of the OMP Regulation, in combination with EU driven funding^1^ and reimbursement at the Member State level, has greatly increased the number of OMPs authorised in Europe and has made OMPs a cornerstone of pharmaceutical markets. Since the year 2000, when the OMP Regulation came into force, the number of annual designation applications has nearly tripled and the number of annual OMP authorisations has increased from only 3 in 2001 to 22 in 2018, see Figure 2.

**FIGURE 3:**
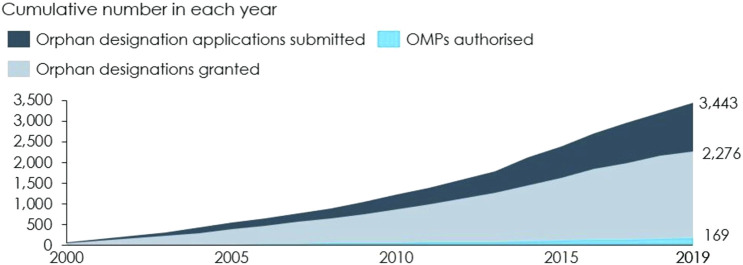
Applications submitted, designations granted and authorised OMPs cumulative. Note: These numbers include applications and authorised OMPs that have been withdrawn. Source: European Commission (2020a) and European Medicines Agency (2020).

Between 2000 and 2019, 3,443 OMP applications were submitted and 169 OMPs were authorised, see Figure 3 (Dolon 2020). Not all of these authorised OMPs can be attributed to the OMP Regulation, but recent estimates indicate that up to 74% of the OMPs authorised between 2000–2017 were developed as a result of the OMP Regulation (Dolon 2020).

Despite the significant increase in authorised OMPs, empirical evidence demonstrates that OMPs continue to represent only a small fraction of EU Member State pharmaceutical budgets - approximately 7% on average. A recent study (Mestre-Ferrandiz et al., 2019) showed that annual per patient treatment costs of OMPs can range anywhere between EUR 755 to over EUR 1 million in the EU. However, approximately 24% of OMPs have an annual cost less than EUR 10,000 and only 18% had an annual cost greater than EUR 100,000—with 58% of OMPs falling between these two thresholds (Onakpoya et al., 2015).

Despite the increase in authorised OMPs, the OMP Regulation has not achieved consistent investment in and development of OMPs. In fact, the needs of rare disease patients in the EU are far from being met.

First, approximately 95% of rare diseases remain without authorised treatment.^2^ In fact, the lack of authorised treatments in rare diseases is broader today than what it was 20 years ago due to the unprecedented rate of newly emerging diseases (European Commission 2020a). It is important to note that this 95% figure does not translate to an equal share of rare disease patients without authorised treatment, as the lack of treatments is particularly eminent for the rarest diseases. Actually, 98% of the rare disease population have a rare disease that is among the 390 most prevalent diseases (affecting 0.1–5 people per 10,000 people) (Wakap et al., 2020). Given the extremely low incidence of some of these diseases it will be impossible to research perform regionally, and globally collaborative efforts are needed (e.g., https://webgate.ec.europa.eu/ern/).

Second, for the 5% of rare disease for which an authorised treatment is available, the treatment is not necessarily transformative**,** i.e., yielding full or partial disease stabilisation, or curative**,** i.e., requiring no further treatment for a period of years (Faulkner et al., 2018).

These outcomes reflect a pattern in OMP development. In the past 20 years, most of the research in rare diseases built on advances in science and on the understanding of diseases. This brings valuable new options, but also leads to clustering of OMPs in certain conditions for which an authorised treatment already exists: of all authorised OMPs between 2000 and 2017, 72% target diseases that have at least one other authorised treatment available. Conversely, only 28% of authorised OMPs target rare diseases for which there is no authorised treatment (European Commission 2020a, 40). The clustering in certain disease areas is not necessarily a problematic development: more innovation and the emergence of multiple treatment options in a specific disease area can benefit patients and meet their therapeutic needs. It also gives healthcare professionals and health authorities larger choice and increases competition in those disease areas. Nevertheless, research and development (R & D) also needs to be directed into those areas where there are no authorised treatments at all.

Understanding this group of diseases with significant lack of treatment, is key to understanding where the challenges with OMP development lie today.

**FIGURE 4:**
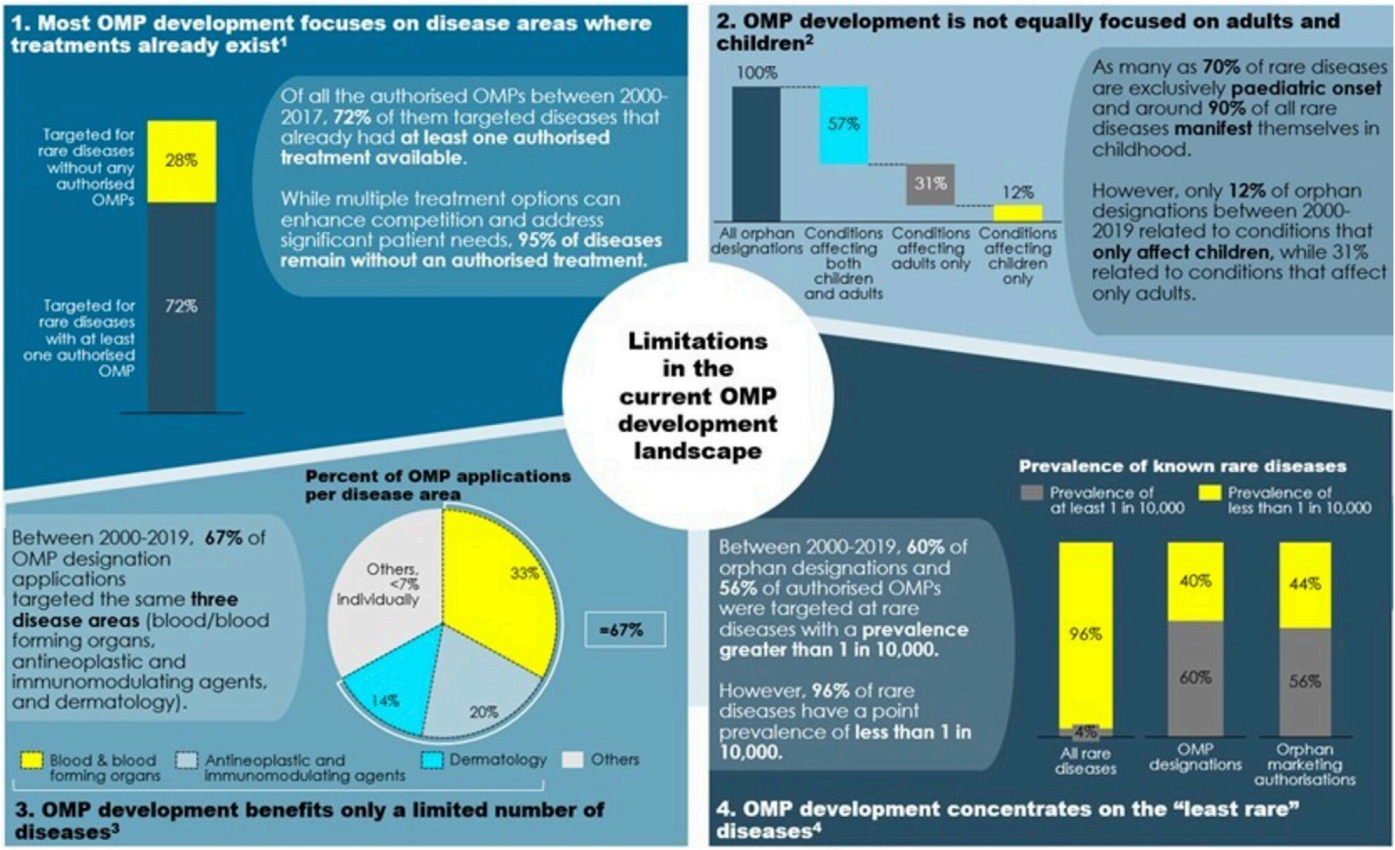
Which areas are concerned by a lack of authorised treatments? Note: 1) Based on authorisations between 2000 and 2017. 2) Based on orphan designations between 2000 and 2019. Source: 1) European Commission (2020, 40). 2) European Medicines Agency (2020, 6). 3) European Medicines Agency (2020, 5). 4) European Medicines Agency (2020, 13–14) and Wakap et al., 2020).

A first look at these diseases (see Figure 4) imposes three preliminary impressions: children with rare diseases have benefitted significantly less from OMP development than adults, OMP development has so far focused on the least rare of the rare diseases, and certain therapeutic areas, such as sensory organs and the respiratory system, have received little attention in R&D so far.

Policy makers’ challenge today is to better understand those areas and to devise a policy framework that delivers continuous innovation in the rare disease space to deliver on patients’ needs for treatment where there is none and for better treatment where treatment already exists.

## The Orphan Drug Expert Group and Its Guiding Principles

Improving the OMP policy framework to address unmet needs is not an easy task as the rare disease environment is both complex and heterogeneous. To manage this complexity, the orphan drug (OD) Expert Group was established in 2020, containing representatives from stakeholders involved in rare diseases drug development, approval and access. The goal of the OD Expert Group was to identify challenges and bottlenecks in the European OMP field and to provide potential solutions. For more detailed information we refer the reader to http://od-expertgroup.eu.

The OD Expert Group worked with sets out four guiding principles that policy makers should follow such that the revision of the policy framework ultimately benefits rare disease patients. These principles have also informed the development of policy proposals by the OD Expert Group itself.

### Conceive a Holistic Policy Framework for the OMP Development Path

Developing OMPs and bringing them to the market is a long path with many stages, from basic research over clinical development to regulatory approval and market access and patient delivery. The development of OMPs can take up to 10–15 years (European Commission 2020a, 13) and challenges with and barriers to OMP development appear throughout the entire OMP pathway.

The current OMP Regulation focuses in on a narrow set of incentives at specific stages of the OMP pathway, particularly clinical development, regulatory approval, and the marketing phase. This creates two challenges.

First, the current Regulation does not provide incentives at all stages where they are needed along the OMP lifecycle. For instance, it provides incentives for the development phase but is not fit to address the lack of basic research that entirely prevents OMP development for some rare diseases. Similarly, the OMP Regulation uses market exclusivity as a main incentive while the main hurdle for many OMPs (especially those indicated for extremely rare diseases) is not the threat of competition *on the market* but making it *to the market* at a price that recovers the investment cost and risk.

The second challenge from this narrow focus is that incentives along the OMP development path are not fully aligned and sometimes even work against each other. For instance, existing basic research may not be development ready due to insufficient guidance of researchers.

Against this background, it is key for EU policy makers to take a holistic look at the entire OMP development path and to design a consistent policy framework that improves incentives for and reduces barriers to OMP development overall.

**FIGURE 5:**
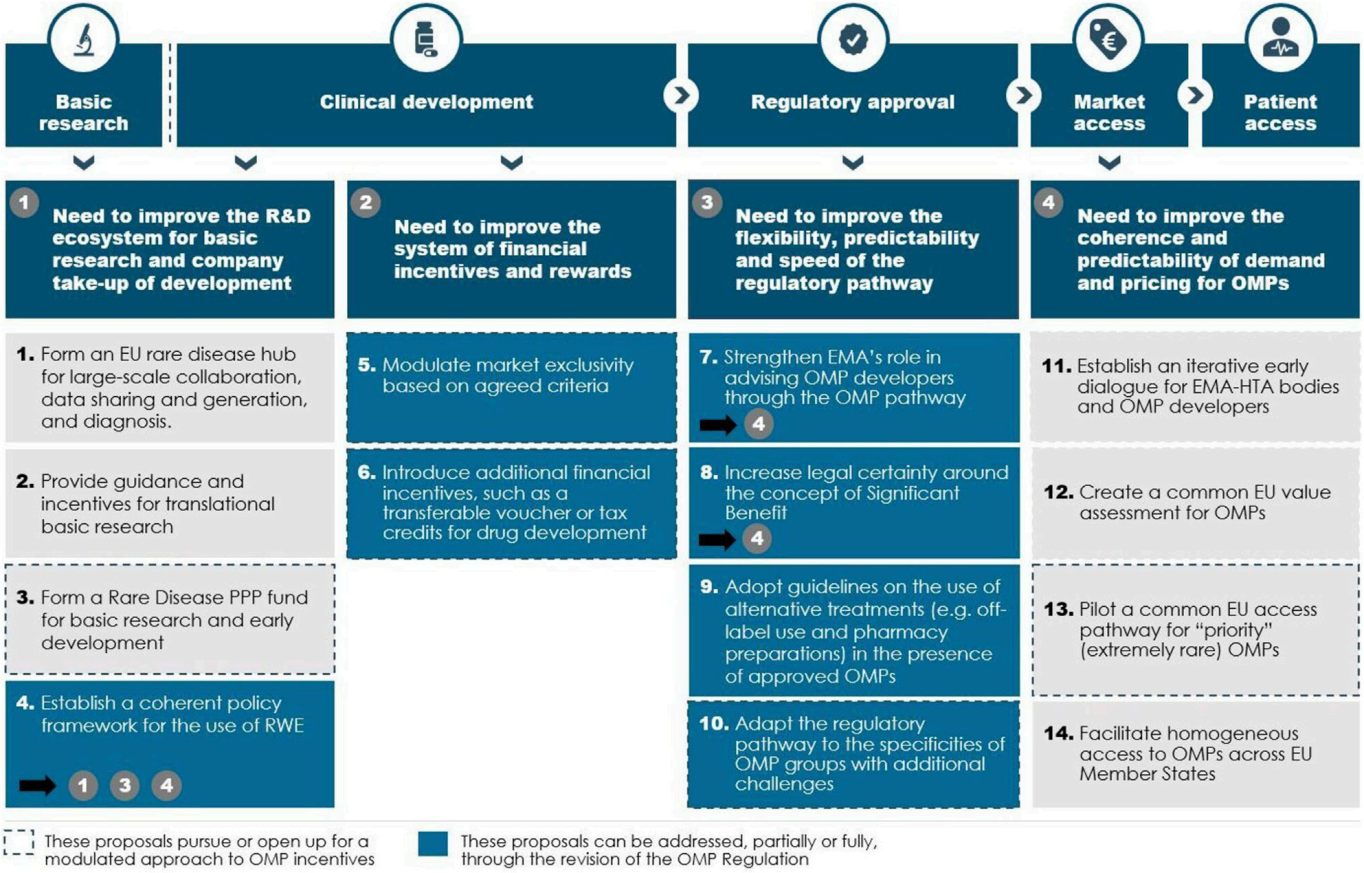
Main needs and policy proposals. Source: The OD Expert Group

This will require wider policy changes beyond the remit of the OMP Regulation and further initiatives under the umbrella of the EU pharmaceutical strategy. The OD Expert Group therefore makes concrete proposals for changes that should be achieved in the current OMP revision and changes that are more long-term in nature (see Figure 5).

### Lead the Revision From a Multi-Stakeholder Perspective

The OMP development path involves many actors: from researchers and clinicians, over pharma companies and funders, to regulators and payers. Most importantly, the path involves rare disease patients and their families who are not only the ultimate recipients of innovative OMPs but also play a role in their pathway through patient advocacy, raising funding for research and participating in clinical trials and other studies.

While all these actors pursue the goal of developing treatments to improve rare disease patients’ lives, they do not collaborate optimally today and lack a strong, unified R&D ecosystem to operate in. One example is in basic research, where collaboration among researchers and between researchers and companies takes place within many, sometimes ad-hoc initiatives. Another example is that HTA bodies, regulators and OMP developers do not coordinate and align sufficiently early enough in the development of OMPs, causing unnecessary delays and uncertainty at later stages. Therefore, an improved OMP policy should strive to strengthen the R&D ecosystem for rare diseases on the one hand and to improve trust and collaboration between the actors on the other. Moreover, any revision should keep in mind the importance of equity and fairness in the treatment of different groups of rare disease patients. To do that, policy makers should adopt a multi-stakeholder perspective in the revision of the policy framework.

### Think About Policy Changes From an Investment Perspective

The EU innovation model builds on a market logic where companies drive OMP development while interacting with all actors in the OMP development landscape: researchers, patients, medical professionals, investors, funders, and regulators. The case for companies to invest in the development of OMPs is, as such, weak due to the high cost and risk in development relative to the low number of patients that OMP developers can achieve revenues on. Companies only engage in OMP development projects if the expected return compensates them for the costs, time and risks incurred in development. Therefore, it is useful to think about changes in the policy framework in terms of their ability to improve investment incentives, see Supplementary Box 1.

**FIGURE 6:**
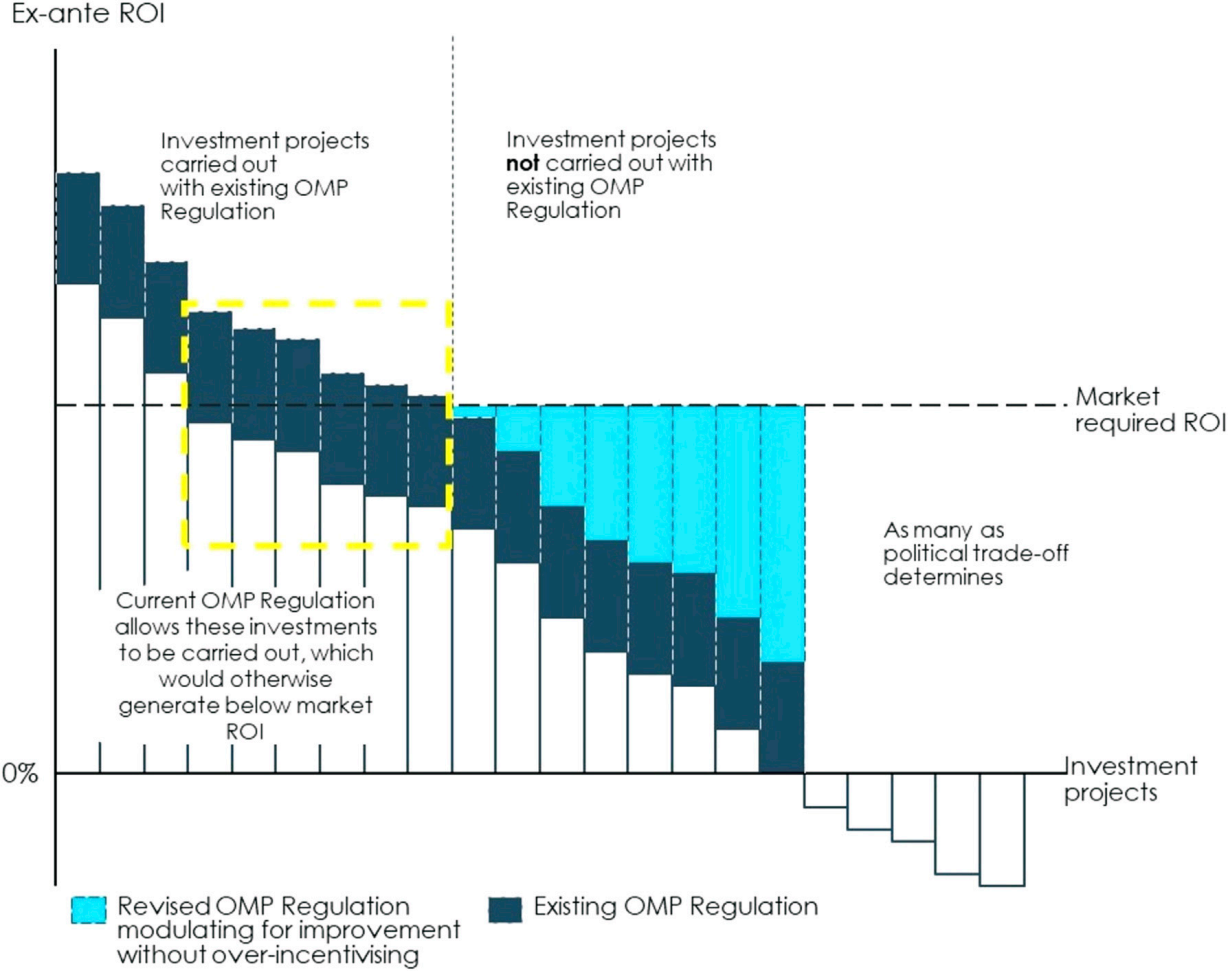
Illustration of how modulated incentives can make OMPs financially viable from an investor perspective. The current OMP Regulation aims to improve incentives by fostering basic research (funding), making OMP development less costly and complex (fee reductions, protocol assistance) and allowing for sales revenues with a lower risk of competition (market exclusivity). In that way, the set of incentives currently included in the OMP Regulation paired with a willingness to pay for OMPs at the Member State level has increased the expected return on investment of OMP development projects, as illustrated by the dark blue bars. However, the lack of approved treatment for many rare diseases shows that there is still a need to strengthen incentives for investing in areas where rare disease patients’ needs are still unmet. To respond to this issue, policies can be designed to improve investment incentives overall. The expected return on investment can be increased through measures that reduce costs along the OMP path, reduce the time it takes for an OMP to go from the basic research stage to market access, increase revenues or set other financial rewards for bringing an OMP to the market. Return on investment can also be improved by reducing the risk of failure throughout the regulatory process and increasing the certainty of market access conditions. Implementing such measures will improve investment incentives overall, i.e., it will expand the yellow box. The current policies provide one-size fits all incentives across OMPs and insufficiently incentivises certain types of projects for which investment incentives are particularly weak. A modulated approach to OMP incentives can provide a level of incentives that is *just* enough to make different OMP development projects (with different investment cases) sufficiently profitable. On the one hand, the current Regulation leaves disease areas where investment projects are not currently carried out**.** These are all projects to the right hand-side of the vertical dotted line. These are cases where the expected return is below what investors can get elsewhere, i.e., the projects for which the dark blue bar is below the threshold of market required return on investment (ROI). There can be diverse causes for an expected return that is too low even at the current policy incentives, such as an extremely small market size or the lack of basic research which makes the project too costly and risky. To address this, the revised OMP Regulation and a revised overall incentive framework (which may include policies beyond the current scope of the OMP Regulation) can strengthen the incentives for as many projects as possible given the political cost-benefit trade-off. These incentives will further increase the ex-ante return on investment reaching the level required by the market, as shown by the light blue bars. Financial incentives or incentives of another nature could be set to target specific categories of OMPs for which the investment case is particularly weak. These could be, for instance, funding for research dedicated to specific diseases with unmet needs or additional years of market exclusivity for specific OMPs. On the other hand, the current Regulation may apply to some OMP projects for which investment incentives are already stronger today than they were 20 years ago thanks to an increase in knowledge in these areas, the existence of both a strong research base and a market for these medicines. For these OMPs (often labelled “crowded areas”) investment incentives are stronger and may even resemble those for non-OMPs (the projects to the left of the dotted-line yellow box). For instance, these could be rare diseases that are close to the prevalence threshold or where the existence of a large body of research and knowledge facilitates OMP development. In these cases, policy makers should find a balance between providing sufficient incentives to ensure continued development of better treatments and softening incentives where they are not necessarily required. Note: Illustrated example. Source: Copenhagen Economics and the OD Expert Group.

The current OMP Regulation aims to improve incentives by fostering basic research (funding), making OMP development less costly and complex (fee reductions, protocol assistance) and allowing for sales revenues with a lower risk of competition (market exclusivity). While those incentives, together with member state commitment to pay for OMPs have increased expected return on investment of OMP development projects, they have not spurred development across all rare disease areas. Therefore, the challenge for the current policy framework is two-fold: first, design the OMP pathway in a way that strengthens investment incentives overall and, second, adopt a modulated approach to incentives with a policy that moves away from one-size fits all to providing a level of incentives that is *just* enough to make different OMP development projects (with different investment cases) sufficiently profitable, see Figure 6.

### Ensure a Competitive EU Policy Framework

The EU policy framework for OMPs does not exist in a vacuum but determines the EU’s perceived attractiveness for funding, developing and launching orphan medicines.

Firstly, to attract OMP funding and investment, the EU needs to provide a competitive policy framework that sets incentives and provides an ecosystem on par with other regions of the world. Currently, this is not the case. The larger number of OMPs brought to the market in the US shows that it is far more attractive to develop and bring OMPs to the market there. For example, between the years 2016 and 2019, there were more than twice as many unique OMPs in the development pipeline in the US than in the EU.^3^ Moreover, most of the investments in gene & cell therapies, the most innovative and promising treatments in the rare disease field, are made in the US.^4^

Secondly, the more aligned the EU regulatory framework is with that of other regions, and in particular, with that of the US, the better the incentives are to register OMPs already registered in those regions in Europe. Recognising that most OMPs are first launched in the US which is the most attractive market in terms of pricing, alignment of EU-US regulations is key. More alignment with the US system, e.g., in clinical trials procedures, will therefore increase the likelihood of OMPs already launched in the reaching European patients more swiftly.

Therefore, even though the OD Expert Group’s recommendations for policy improvements focus on Europe, the importance of the international context must not be forgotten.

## The Policy Proposals

### Four Needs for the EU OMP Incentive Framework

From discussion sessions amongst the members, it became clear to the OD Expert Group that the barriers to and challenges with OMP development appear throughout the OMP development path. Based on the experts’ experiences with different stages of the OMP development path, the OD Expert Group identified four broad needs for OMP development in the EU today:

1) The need to improve the R&D ecosystem for OMPs to increase the scale and scope of basic research and company take-up of clinical development.
2) The need to improve the system of financial incentives and rewards to improve the investment case for developing OMPs in priority disease areas, such as disease areas without authorised treatments.
3) The need to review and improve the flexibility, predictability and speed of the regulatory pathway for OMPs to better accommodate for the unique needs of rare disease development projects.
4) The need to improve the coherence and predictability of demand and pricing of OMPs to integrate and align demand-side incentives with the overall OMP incentive framework.

Delivering against the four needs will lead to an improvement of the incentives for OMP development in general and for areas without authorised treatment in particular.

As a potential solution, the OD Expert Group makes 14 policy proposals that allow to serve those needs. The proposals aim at improving incentives for OMP development *overall* by removing barriers in the current policy framework or by making better use of current initiatives and expertise. Therefore, the proposals build as much as possible on existing policies, structures and initiatives in the EU OMP space.

Moreover, the proposals follow the idea of a more modulated approach to OMP development reflecting the heterogeneity of the rare disease landscape.

Modulation means offering tailored incentives to reflect the investment case for different OMPs and requires a differentiated understanding of the investment case for different sub-groups of OMPs. Modulation to meet unmet needs requires setting additional incentives for specific groups of OMPs where, currently, insufficient incentives exist. While the identification of a modulation mechanism is beyond the scope of this report, we refer the reader to Box 2 for a more in depth discussion.

Together, the set of policy proposals jointly optimise development incentives along the OMP drug development path, thereby allowing for more OMPs to be developed faster across the EU. The proposals both aim to improve the incentives for developing more *effective* treatments and developing treatments where *none exist today*, see Figure 5.

### Need 1. Improving the R&D Ecosystem for Basic Research and Company Take-Up of Development

Basic research by academics and clinical development by companies are the backbone of OMP development. All drug development relies on basic research, as without understanding of underlying disease mechanisms, biomarkers and targets, it is impossible to develop responsive treatments. In recent years, innovative research methods have led to successes in offering better, quicker and easier identification of, for instance, the genetic origins or rare diseases. Examples of this are whole-exome sequencing (WES) and whole-genome sequencing (WGS) (Liu et al., 2019; Posey 2019), which have led to great success in the speed and precision of which a range of genetic rare diseases are diagnosed.

However, the lack of treatments is also broader today than what it was 20 years ago, due partly to better identification and sub-grouping of known rare diseases and treatments, but also due to the emergence of new diseases (European Commission 2020a). Hence, notwithstanding the successes of OMP development in the last 20 years, many rare diseases today continue to lack very basic research and understanding of underlying disease mechanisms. In other words, for many rare diseases, the scientific base from which drug development can depart from is either non-existent or insufficient.

There are four main reasons behind the shortage of research and company take-up of clinical development in the rare disease space. First, the 6,000–8,000 known rare diseases cover a broad plethora of syndromes, but with many commonalities. This leads to delays and difficulties in diagnosis, and often culminates in misdiagnosis. Without timely and accurate diagnosis, it can be difficult to collect patients for studies. It takes on average 8 years (EURORDIS 2021a) to diagnose rare disease patients, during which time the patient and societal burden grows to be significant.

Second, the patient populations for individual rare diseases are small and geographically dispersed - particularly among the rarest diseases. This means that it is not only difficult to identify and diagnose patients but also to study rare diseases in pre-clinical and clinical settings, and any available knowledge and data is typically held by a few and geographically dispersed specialists and research institutions. This knowledge is not effectively clustered because researchers, companies, patient groups and clinicians do not collaborate sufficiently across the rare disease space, leading to insufficient scale in research.

Third, although a substantial amount of research is already happening in Europe, it is often not mature enough for drug discovery and further development, i.e., it is not translational research.

Fourth, it is difficult to find and secure funding for not only the basic research itself, but also for translating it into development-ready research. The challenges lie in the level and the cohesion of European rare disease funding efforts—where, in addition to the funding coordinated by the European Joint Programme for Rare Diseases (EJP RD), further financing is required to truly scale up the European R&D ecosystem for rare diseases.

If the R&D ecosystem is not improved, existing research may continue to remain unexploited for drug development - because opportunities for scale are missed or because data and knowledge are not transmitted between different stakeholders.

These challenges impose a clear need to improve the R&D ecosystem for basic research and company take up of clinical development. The European R&D ecosystem needs better financing and collaboration infrastructures, geared towards pursuing the unique challenges and policy goals of conducting research in rare diseases—and particularly in areas where no or little research exists. Moreover, the R&D ecosystem should be easy for researchers, OMP developers and funders to navigate, such that resources are findable, accessible**,** interoperable and reusable (FAIR) across different rare disease projects (Wilkinson et al., 2016).

To improve the R&D ecosystem, the OD Expert Group makes four policy proposals. These four proposals are designed on the basis of existing initiatives in rare disease research and should therefore seek to connect and build upon the existing work.

#### Proposal 1. Form an EU Rare Disease Hub for Large-Scale Collaboration, Sharing and Generation of Data, and Diagnosis

**FIGURE 7:**
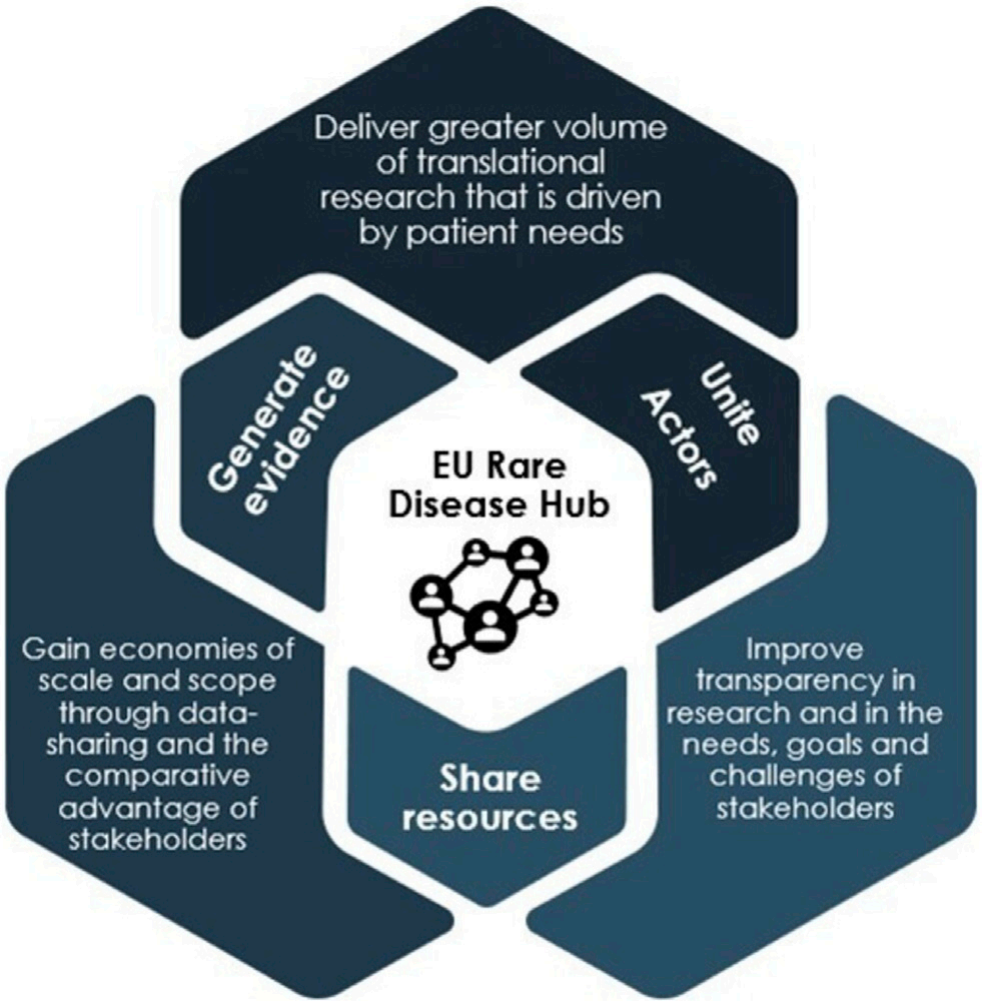
Eu rare disease hub. Source: The OD Expert Group.

Since the first European Reference Networks (ERN) were launched in 2017, the EU has taken great steps in improving the exchange of information and expertise in rare diseases. However, today, scientific knowledge on rare diseases is still scattered across different European institutions and initiatives, and unavailable to many important actors. In a fragmented ecosystem, the full potential of the existing and potential European research efforts is not reaped. A crucial step in unifying rare disease R&D is therefore to establish a collaborative EU rare disease hub, which builds upon the ERN infrastructure, as a one stop-shop for collaboration between all actors in the sharing of knowledge, generation of new evidence, and in diagnosis. The hub will become the central infrastructure connecting all scientific knowledge on rare diseases in Europe serving two main purposes (Figure 7).

First, the hub provides for greater and more consistent, systematic collaboration between researchers, companies, clinicians, patient groups and other actors in R&D—both within and outside of Europe. By bringing rare disease basic research, much of which currently exists in silos, to a single platform, the hub enables the actors involved in rare disease R&D to gain an overview of areas in which research is taking place, identify areas of collaboration and also determine areas which remain entirely unaddressed. Thereby, the hub will.

• Enable coordination of research efforts and a more optimal use of resources through grouping diseases
• Enable faster and broader take-up of clinical development through signalling areas of development-ready research to companies and investors
• Allow basic research to be better aligned with clinical development and patient needs early on.

As a coordinating body, the hub can also facilitate collaboration in both the mapping of patient populations and in the diagnosis of rare diseases. Collective, coordinated mapping of patient populations is a precondition for improving our understanding of the incidence of rare diseases across Europe. Similarly, harmonised diagnosis is more effective than current national diagnosing practices, as it harnesses existing and scattered expertise in a more coordinated manner, and thereby create more scale in diagnosing patients.

One initiative that the hub could coordinate is Newborn Screening (NBS)**,** which is (for various rare diseases) currently performed nationally across the EU. The hub could facilitate harmonised NBS programmes across Europe, following EURORDIS’ Key Principles for Newborn Screening (EURORDIS 2021b).

Second, the hub will enable better exploitation of existing rare disease data through a common data infrastructure, where the generation, sharing and use of key data, including traditional clinical and preclinical data and real-world evidence (RWE), between stakeholders can take place. With current data existing largely in scattered databases in different formats, a main advantage of the hub is the centralisation and standardisation of data to make existing and new data more findable, accessible, interoperable and reusable across different rare disease projects. This would enable wider and quicker access to important data for all stakeholder groups and facilitate the collection of treatment candidates from existing research, thereby de-risking and speeding up OMP development.

A common data infrastructure will also facilitate the exploitation of existing knowledge and the adoption of new, advanced digital data technologies, including Artificial Intelligence (AI). This will allow, for instance, for existing innovative diagnostic methods to be repurposed and improved. It will also enable scale in diagnosis and in grouping of diseases**,** thereby enabling a basis for prioritisation and potential modulation of incentives (see Figure 5)**.** This is of particular importance for very rare diseases, where innovative diagnosis can identify patient populations more effectively and disease grouping will facilitate knowledge sharing among researchers and clinicians.

The hub can connect and build on many existing EU-wide R&D initiatives and structures in place today. The efforts of the hub can exist under the umbrella of EJP RD, which is already leading European initiatives for large-scale collaboration and data sharing. Notably, the hub should connect, and build on, the structures and expertise within the 24 existing rare disease ERNs (Heon-Klin 2017). The hub can also build on the RD Connect project^5^, the EJP Virtual Platform^6^ led by the EJP RD, and the EU RD Platform^7^, created by the Commission’s Joint Research Centre, by making the data accessible to all stakeholders.

To be feasible, the EU rare disease research hub will need to be accompanied by incentives for the sharing of data. For instance, rare disease funding could be made conditional on data-sharing or open-source publication.

#### Proposal 2. Provide Guidance and Incentives for the Translation of Basic Research

Where rare disease basic research is taking place in Europe, it is often not developed enough to enter the clinical development stage. Preclinical studies, such as proof of safety, are crucial in determining whether a drug will proceed to human studies and how subsequent trials should be designed. Therefore, the produced research needs to be translational**,** i.e., enable industry to translate the basic research into treatments for patients without incurring a prohibitive level of uncertainty or delay.

This requires common guidelines for how translational research and a framework with appropriate incentives for producing development-ready research should look. Guidance on clinical preparedness can come, for instance, from the Orphan Drug Development Guide prepared by the International Rare Diseases Research Consortium (IRDiRC), an organisation that has already taken multiple actions to support translational research in the rare disease space (Jonker et al., 2020).

Making research funding conditional on producing development-ready research could be an effective incentive for researchers. This will make the generation of development-ready research a standard procedure for the rare disease basic research community, but also ensure the relevance and usability of the knowledge along the innovation cycle.

#### Proposal 3. Form a Rare Disease PPP Fund for Basic Research and Early Development

Today, EJP RD leads the most systematic and coordinated funding efforts for rare disease basic research in Europe. However, generating sufficient research to address unmet needs requires the EU to increase the scale and continuity of funding for basic research and early development above and beyond the duration of the EJP RD.

A way forward is to establish a singular financial entity, a basic research private-public partnership (PPP) fund, where the financial responsibility of serving more rare disease patients with effective treatments is mutually shared by public and private financing sources. Such a fund will improve the financing infrastructure for OMPs at large by generating 1) more funding and 2) more directed and conditional funding.

First, more funding can be achieved by incorporating more actors in the financing structure.

Alongside EU and national-level funding programmes (financed by tax revenues), the public funding side of such a fund should incorporate for instance the European Investment Bank (EIB), which is already investing in the rare disease space and other important health initiatives, such as Global Fund (Dorozynksi 2003).

In order to sustain the sustainability of public budgets, pharmaceutical industry actors (both OMP and non-OMP developers) need to be integrated in the coordinated funding structure as a key financing source. Contributing industry actors should not be eligible for funding, but rather, would benefit indirectly from collaborating in the projects, e.g., *via* in-kind contributions and for contributing to project descriptions. In this way, the capacity of smaller actors, such as SMEs, can be increased to undertake R&D in rare diseases, while the (larger) industry actors are still incentivised to contribute.

In addition, the Rare Diseases PPP fund could coordinate with European life sciences-focused Venture Capital (VC) in an effort to attract VC presence in rare disease research and facilitate early-stage development. However, this should include measures that incentivise the investment of VC firms in riskier early-stage projects. The PPP fund should provide transparency and trust in potential long-term growth, e.g., with dedicated investment specialists possessing required scientific knowledge.

Second, more directed and conditional funding can be steered by an appointed governing board, which would be responsible for ensuring that the strategic goals and research objectives of the fund are aligned with the unmet needs of patients. The governing board could be jointly coordinated by EJP RD, the European Commission (EC), EMA as well as industry organisations (European Federation of Pharmaceutical Industries and Associations (EFPIA) and European Confederation of Pharmaceutical Entrepreneurs (EUCOPE)), in order to ensure both balanced representation and rare disease knowledge.

The advantage of such a coordinated, top-down setup is that it can efficiently direct funding towards selected avenues, such as specific disease areas. This can offer diseases without sufficient patient group support, such as many of the rarest diseases, a more equal chance of being picked up for research and development. In addition, this setup can also impose certain conditionality on funding, in particular regarding the quality and outcome of the research.

For example, funding could be conditional on producing development-ready research and on sharing data with the wider OMP research community.

A broader operating framework needs to be established for the fund, e.g., by the EC, including specifications on the level of freedom and constraints that different funders can operate with, the financing terms, overall governance and use of resources. The governing board could act as a scrutiny board, assessing and providing guidance on budget use and procedures, thereby ensuring that funds are allocated efficiently and effectively.

Lastly, the Rare diseases PPP fund should operate closely with the proposed EU Rare disease Research Hub in order to ensure funding is directed towards the needs of patients and the seamless transferability of knowledge and data between the two bodies.

#### Proposal 4. Establish a Coherent Policy Framework for the Use of RWE

RWE is evidence on the usage and potential benefits or risks of a medical product derived from analysis of (real-world) data. RWE is particularly relevant for the OMP development pathway due to the higher hurdles OMP developers face in collecting sufficient evidence in more standard clinical trial settings. RWE can therefore be an important input into R&D, regulatory approval and decision-making on pricing and reimbursement at the market access stage.

However, today, the potential of RWE at all stages of the development path is underexploited because they are not integrated and recognised in regulatory decision making and because the lack of harmonised standards and guidelines results in mistrust towards such evidence, see Supplementary Box 1.

In particular, the role of RWE can be enhanced at three stages of the development path: the R&D stage, the regulatory approval stage and the market access stage.

##### Enhancing Access and Standardising RWE to Facilitate Rare Disease Research

Systematic collection of and infrastructure for sharing RWE between stakeholders can facilitate research on rare diseases. This can be part of a larger effort to better exploit existing data and more effectively generate new knowledge in the proposed EU Rare disease Research Hub.

##### Better use of RWE to Improve the Evidence Base at the Regulatory Approval Stage

RWE improves the chances of regulatory success of OMPs by bridging the gap between evidence collected through clinical data and regulatory requirements. Establishing a consistent framework for the utilisation of RWE will maximise its role across the various stages of regulatory development for OMPs, thus derisking the development without lowering the evidentiary standard.

##### Better use of RWE to Improve the Evidence Base at the Market Access Stage

OMP developers often struggle to gather enough traditional clinical evidence to prove the relative therapeutic value of an OMP at the market access stage. While there may be sufficient data from the clinical trials to support a positive benefit-risk assessment and a full, or conditional, marketing authorisation, there may be a lack of data to support clinical effectiveness in the stringent value assessments of payers and health technology assessment (HTA) bodies. Failure at the market access stage is in fact often linked to perceived deficiencies in the evidence collected on safety, efficacy and additional benefit compared to existing treatments. Structured presentation RWE should therefore serve and be recognised as a complementary form of evidence in those assessments.

### Need 2. Improving the System of Financial Incentives and Rewards

Financial incentives and rewards are monetary benefits offered to encourage behaviour or actions which otherwise would not take place. Next to the price offered at the market access stage, financial incentives are the most direct way of incentivising OMP development. In practice, financial incentives can act both on the cost-side, reducing costs for OMP developers, or on the revenue-side, allowing OMP developers a sufficient return on their investments.

Currently, the OMP Regulation foresees two types of financial incentives: 1) fee reductions in the regulatory phase to reduce OMP developers’ overall costs in bringing OMPs to the market and 2) a 10-year period of market exclusivity at the time of receiving marketing authorisation, which protects OMP developers from competition from similar medicines thus ensuring a sufficiently high level of revenues to recoup investments and remunerate the risk taken.

The fact that 95% of rare diseases remain without authorised treatment suggests that the current financial incentives are not sufficient to steer development into areas of unmet need. In particular in disease areas with a very limited number of patients, protection from competition of similar drugs may not act as a strong incentive, because competition is not the main concern for OMP developers. Instead, the concern not to get market access at a sufficient scale and price may deter OMP developers from investing.

A well-designed set of targeted financial incentives will work in conjunction with the improved R&D development ecosystem to encourage development to address specific (priority) diseases. The new or improved financial incentives can be modulated in such a way that they encourage investment in priority diseases, while still incentivising continued research across all rare disease areas.

The OD Expert Group identifies two financial incentives, which can be used as tools to improve the investment case for areas of greatest unmet need.

#### Proposal 5. Modulate Market Exclusivity Based on Agreed Criteria

Market exclusivity is an important incentive of the OMP Regulation that delays the permission for other companies to produce generic drugs with the same mechanism of action for the same indication. This allows an OMP developer to generate revenues and recover investments in a market free from competition from similar drugs (with similar indications). However, market exclusivity does not preclude developers from developing other drugs for the same indication. As a way to bring more aligned incentives into a heterogeneous market, the OMP Regulation can use market exclusivity as a modulation tool to attract development into priority disease areas, while keeping incentives for developing OMPs in other areas equal at the margin. In practice this means that market exclusivity for OMPs addressing defined priority diseases would be extended beyond the standard period of 10 years. A longer exclusivity period offers an opportunity to generate higher revenues for a longer period, which can be particularly useful for very rare and slowly progressing diseases where more patients can be covered during the period. Conversely, as a way of balancing incentives, the market exclusivity could also be shortened as a way to soften policy incentives in areas where development incentives are already strong.

The exact design for how to modulate market exclusivity requires a thorough, and separate, assessment, in order to ensure that incentives are fair and yield optimal outcomes across OMP projects. In addition, such modulation would require a consistent framework for the identification of “priority diseases”.

Alternatively, market exclusivity can be used to incentivise behaviours which benefit the EU rare disease R&D ecosystem. For instance, the generation and sharing of (commercially valuable) data, such as RWE, could be rewarded through an extended exclusivity period. This would ensure that there is an incentive to share important data across the rare disease R&D community, thereby facilitating knowledge sharing and the development of effective therapies.

#### Proposal 6. Introduce Novel Financial Incentives, Such as a Transferable Voucher or Tax Credits for Drug Development

**FIGURE 8:**
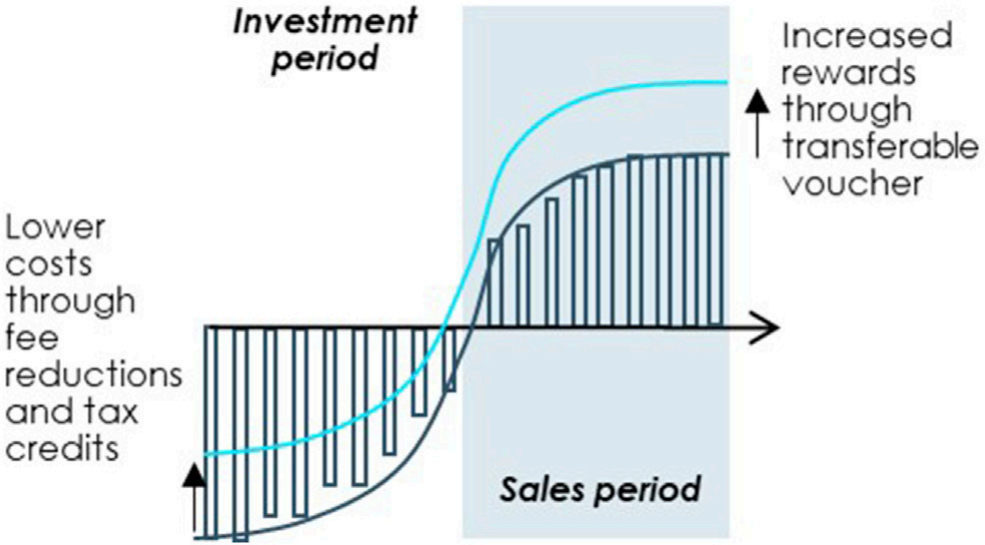
Improving the OMP investment case through targeted financial incentives. Note: Illustrated example. Source: Copenhagen Economics and the OD Expert Group.

Additional financial incentives are a useful way of steering development into priority areas provided that they are carefully designed to achieve favourable outcomes for society at large. For the incentives to be relevant for OMP developers, they should either decrease costs during the investment phase or increase rewards at the time of market access, see Figure 8.

The OD Expert Group offers two examples of financial incentives that can be devised to steer R&D into specific rare diseases by increasing market phase rewards or decreasing costs for OMP developers: 1) a transferable voucher and 2) fiscal incentives for drug development**.** The detailed design and introduction of these exemplary financial incentives should be supported and preceded by an impact assessment.

**Example 1**: A transferable voucher

A transferable voucher can be used as a targeted market-driven incentive for directing investments into priority diseases. The innovation behind a transferable voucher is that it awards the developer of a new medicine in a specific priority disease with a voucher for some additional rewards for a future (orphan or possible non-orphan) medicine in their portfolio, or to be sold on the market to other medicine developers.

The transferability of the voucher ensures that it is an incentive not only for larger OMP developers with both rare disease and blockbuster medicines, but for smaller rare disease-focused companies, foundations and academic institutions that can sell their priority vouchers to fund additional research in the rare disease field. This ensures allocative efficiency, resulting in a more dynamic and efficient secondary market for OMP development.

There are three primary design considerations that need to be taken into account in order to ensure feasibility and effective and efficient outcomes.

First**,** a primary consideration is who should be eligible for the vouchers. The recipients should be those that have the scientific expertise and capability to develop OMPs for specific rare diseases, but otherwise lack the financial means or commercial viability to do so. The mechanism for selecting voucher recipients, as well as diseases to be prioritised, should be established by a governing body, e.g., through the EMA, and could take inspiration from the US voucher system.

Second**,** there are several ways in which a voucher can reward OMP development. For example, the EU could consider any of the following rewards:

• Accelerated regulatory review (similar to US Rare Paediatric Voucher), awarding the selected portfolio drug with quicker regulatory process and market access. It is important to note, however, that such a reward may direct finite regulatory resources away from processing the applications of more important drugs in the future, such as OMPs, to the detriment of patients with potentially no treatment options (Mezher et al., 2020).
• Extension of market exclusivity**,** delaying generic competition for any future portfolio drug. This would improve the potential returns that the voucher holder could achieve on the market, without requiring as many regulatory resources from the EMA. However, this reward should entail certain monetary and time caps, as to ensure fairness to generic manufacturers and national health budgets (Outterson and McDonnell 2016).
• Automatic access to the PRIME^8^ scheme**,** awarding a future drug with all PRIME scheme benefits. This requires that the future drug, OMP or non-OMP, is eligible for PRIME scheme, but it also ensures that future regulatory resources are more efficiently spent on more critical treatments than, for example, blockbuster drugs.

Third, the voucher holder should be obliged to market the OMP for which the voucher is awarded for. This would require that any transferability is not possible until the OMP is authorised (or marketed in at least one Member State). The EMA should hold full rights to reclaim the voucher, should the original voucher holder fail to market the OMP.

**Example 2**: Tax credits for drug development

Reducing development costs will improve the investment case for OMP development. Fiscal incentives, such as tax credits, allow OMP developers to save costs as a result of intense R&D activity. In the US the Orphan Drug Tax Credit (ODTC) is designed to promote research spending on OMP development, granting developers a 50% tax credit of clinical trial costs for OMPs.

Since clinical trial costs alone are a large part of the overall drug development costs, this instrument would increase the likelihood of more OMPs advancing from basic research to clinical development in Europe. Similarly, as this would lower the cost barrier to conduct clinical trials in Europe, we could see a more equal share of clinical trials being conducted in Europe and the US, thereby creating a more vibrant R&D ecosystem for OMPs. A 2015 study on the US incentive estimates that approximately one third of drug development investment in the US is attributable to the ODTC (NORD 2015, 22).

Direct application of tax credits to Europe might pose some challenges as taxes are a national competence. However, it is possible to mimic similar incentives by creating a designated European fund to be shared between companies that conduct research for OMP development in Europe. The feasibility of such an initiative is outside the scope of this exercise and should be further investigated in a separate study.

### Need 3. Increasing the Flexibility and Predictability of the OMP Regulatory Pathway

The term “regulatory pathway” refers to the set of steps required for the regulatory approval of OMPs. The characteristics of the regulatory pathway influence costs, time to market and risk of OMP development projects. As a result, they influence the number of OMPs that reach patients and the speed with which they do so. A regulatory pathway that is not sufficiently flexible or predictable results in costlier, more time consuming and riskier OMP development projects.

**FIGURE 9:**
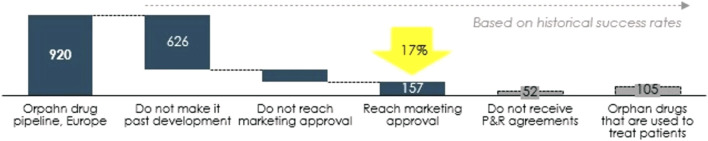
From pipeline to orphan drugs accessible to patients, number of OMPs. Source: Copenhagen Economics based on EMA data (European Medicines Agency 2020).

One of the problems concerning OMPs is the high rate of attrition along the development path. Only around 17% of OMPs reach market approval and even fewer succeed in pricing and reimbursement negotiations, see Figure 9. A well-designed regulatory pathway that addresses the specific challenges of OMP development can, in combination with other measures, contribute to a lower attrition rate. Therefore, EU policy makers should shape the regulatory pathway to ensure high flexibility and predictability of OMP development.

Firstly, the regulatory pathway needs to be sufficiently flexible both in terms of ways in which OMP developers can meet the standards of evidence and in relation to the interaction between parties involved. OMP developers may have difficulty in producing sufficient evidence in the traditional clinical trial setting. This is due to small and dispersed patient populations and slowly progressing rare diseases, making the use of conventional clinical endpoints not always possible or efficient (McCune 2017). A regulatory pathway that is flexible to different types of evidence, without lowering the evidentiary standards, will contribute to reducing the costs, risks and time to market for OMPs.

The interactions between OMP developers and regulatory bodies could also benefit from additional flexibility. For instance, the standard advice framework with the EMA may appear rigid in some instances, with limited opportunity for flexible dialogue. This leads to a situation where OMP developers may not receive support and guidance when they most need it. More flexible interactions ensure timely guidance, and in turn, faster, less risky and possibly less costly OMP development, provided that the advice is implemented in the development plans. Flexibility is not only useful in improving the regulatory pathway for all OMPs but also for accommodating the specific needs of sub-groups of OMPs. Certain sub-groups of OMPs face additional challenges across the development path. For different reasons, the regulatory process becomes slower, more costly and riskier. A flexible pathway that can be tailored towards the specific needs of these sub-groups will improve the investment case for these OMPs.

Secondly, predictability is essential to maximise the benefits of the incentives provided by the OMP Regulation. Currently, certain aspects of the regulatory pathway are not sufficiently predictable, thereby adding unnecessary risk to OMP development. This largely stems from the fact that OMP developers face overlapping and inconsistent requirements from the different authorities (the EMA, HTA bodies and payers) across the development path. For example, although legislative provisions provide examples (European Commission 2000; European Medicines Agency 2009), there is still high uncertainty on the type and level of evidence required by the Committee for Orphan Medicinal products (COMP) in proving significant benefit to obtain and maintain orphan drug designation (ODD).

This problem is particularly pronounced at the time of confirming ODD when indirect comparisons must be made (in the absence of clinical evidence), for which there is currently no agreed standard methodology (Fregonese et al., 2018; Nicolodi 2019). In addition, confirmation of ODD is required when the therapeutic indication is significantly broadened and may also be required 5 years after obtaining the market authorisation. Each time the ODD requires confirmation, newly approved products are taken into account in proving significant benefit, increasing uncertainty.

A further example is that of conditional marketing authorisation, where the lack of data is accepted at the regulatory approval stage but often leads to difficulties in negotiating pricing and reimbursement at the market access stage (Malinowski et al., 2018).

Increasing certainty and consistency of processes across the development path will reduce the perceived risk, cost and time and improve the ex-ante investment case for investing in developing OMPs, maximising the potential of the incentives provided by the OMP Regulation. This requires that there is alignment between the different authorities, such that consistency can be achieved also beyond the regulatory stage.

The OD Expert Group puts forth four policy proposals for improving the flexibility and predictability of the regulatory pathway for OMPs. These proposals are designed with the challenges associated with the processes and requirements for obtaining regulatory approval for OMPs.

#### Proposal 7. Strengthen EMA’s Role in Advising OMP Developers Through the OMP Pathway

The EMA is an important actor for European OMP developers and oversees the regulatory pathway for the entire lifecycle of an OMP, from initial orphan designation through marketing authorisation to post-licensing. The EMA provides guidance and opportunities for interaction in the development phase as well as guidance and timelines for each step of the regulatory pathway. However, the current collaboration model between EMA and OMP developers is perceived as rigid, with limited opportunities for dialogue and underutilisation of the guidance that the EMA can offer. Strengthening EMA’s role as an advisory body for OMP developers and thereby improving cooperation is a way to flexibly adjusting the regulatory pathway to the needs of individual OMP development projects and to ensure that the EMA is best equipped to guide OMPs towards regulatory approval. Two steps are needed to achieve this goal:

The first step is to establish an iterative advice framework, for both regulatory and scientific advice, where OMP developers can receive the EMA’s advice and guidance on a more consistent and less formal basis—both in the approval process and early on in parallel to drug development. Implementing this will likely require additional resource for the EMA. In practice, an iterative advice framework could supplement the existing PRIME scheme^9^, which is in place for selected priority medicines, by increasing the coverage and frequency of advice to all rare disease projects.

The second step is to strengthen the COMP and improving alignment between the COMP and the Committee for Human Medicinal Products (CHMP). The role of the COMP is crucial because it is the body within the EMA that better grasps the hurdles of OMP development. Therefore, the COMP should be endowed with sufficient resources and experts to ensure that the regulatory pathway is best suited to guide OMP developers. The role of the COMP should also be strengthened within the EMA such that it can follow OMPs throughout all the stages of the regulatory pathway. Finally, ensuring alignment between the COMP and the CHMP throughout the different stages will reduce the risk of frictions and enhance predictability. For instance, ensuring alignment between the guidance provided by COMP and the scientific advice provided by the CHMP will improve predictability.

#### Proposal 8. Increase the Legal Certainty Around the Concept of Significant Benefit

Significant benefit plays a role at two stages in the regulatory process: the initial stage is when a medicine developer submits an application for orphan designation early on in a medicine’s development, Significant Benefit is then often assessed based on assumptions since most products at the time of ODD will be at preclinical or early clinical stage of development.

Subsequently, Significant Benefit needs to be confirmed at the time of marketing authorisation based on a thorough comparison with all OMPs approved up to that moment in time. In addition, Significant Benefit has to be demonstrated at the time of marketing authorisation (MA) irrespective of the type of MA (e.g., there are no special provisions for a “conditional” Significant Benefit in cases when the product receives a conditional MA). While the concept of Significant Benefit ensures continuous innovation to the benefit of patients, it lacks legal certainty and predictability that introduces unnecessary risk in the OMP development path.

Firstly, the concepts and scientific contents of Significant Benefit and the type and level of evidence required for its demonstration are not sufficiently clear, especially when only indirect comparisons are available.

In addition, the current regulatory framework is inconsistent as it provides for the possibility of a conditional MA in advance of providing full evidence but still requires full proof of significant benefit. In situations where an OMP developer is unable to provide comprehensive safety and efficacy data at the time of MA, and is therefore granted a conditional MA, the level of evidence is unlikely to be enough for the Significant Benefit assessment. This means that an OMP may be granted conditional marketing authorisation but may lose the Significant Benefit status and the ODD, thereby causing high uncertainty on future revenues.

Therefore, there needs to be more alignment in the evidentiary standards required for the Significant Benefit assessment and for MA—ideally by a “conditional” Significant Benefit status, where evidence for proving significant benefit would continue to be provided post-MA. The application and feasibility of this should be explored further, as it is outside the scope of this report.

Secondly, OMP developers may have considerable difficulty demonstrating Significant Benefit compared to OMPs that obtained MA close in time to the re-assessment. This may create uncontrollable risk in the OMP development pathway.

Thirdly, the recognition of Significant Benefit at the regulatory approval stage does not necessarily carry over into the value assessment at market access stage. This brings uncertainty on market access conditions and duplication of costs and time at the market access stage.

These challenges call for an improvement of legal certainty and predictability of the Significant Benefit concept. Three concrete steps can help achieve this goal:

First, the concept of Significant Benefit can benefit from clearer and more transparent guidance, particularly in the case of indirect comparisons. A higher level of certainty can be achieved through 1) clearer and more transparent guidelines and 2) closer cooperation on a case-by-case basis between the OMP developer and the COMP in defining the data requirements early on. Enhancing the role and use of the existing scientific advice framework can be a step in this direction and this is an example of where the iterative advice framework with the EMA will be beneficial. Clearer guidance should also align the concept of Significant Benefit with that of conditional marketing authorisation.

Second, the risk of companies’ failure to prove Significant Benefit at the approval stage can be significantly reduced by restricting the comparator treatments to those OMPs with a marketing authorisation granted at least 1 year prior to filing the marketing authorisation application for the non-similar OMP. This will ensure that OMP developers know in advance which products will be considered and have sufficient time to collect the required data to meet the evidentiary standard.

Third, where Significant Benefit is recognised at the regulatory approval stage, it should be recognised as an ‘added value’ of the OMP in question at the market access stage. The European Commission decision certifying the presence of Significant Benefit compared to other approved treatments provides useful information for the national value assessment of the OMP. In practice, national HTA bodies and payers should recognise the European Commission’s decision and reflect the presence of Significant Benefit in determining the value of OMPs and in market access conditions, specifically with reference to price benchmarking with comparators, see Supplementary Box 3.

Recognising the assessment of Significant Benefit at the regulatory stage in the value assessment at.

The market access stage will bring certainty and reduce duplication of costs and time spent. It will also create a continuum in the value assessment and perception along the OMP development path.

In addition to these proposals, the OD Expert Group urges EU policy makers to take stock in 10 years’ time of the advantages and draw-backs of the Significant Benefit concept and to re-assess its usefulness as part of the regulatory framework.

#### Proposal 9. Adopt Guidelines on the Use of Alternative Treatments (e.g., Off-Label and Pharmacy Compounding Preparations) in the Presence of Approved OMPs

OMP developers expect that after maintaining the ODD at the time of marketing authorisation.

They will benefit from 10 years of protection from competition from similar products (for the same indication). Challenges to the market exclusivity cause uncertainty and increase the risk associated with OMP development. Such challenges currently come from unclear rules around the off-label use of medicines, hospital exemptions and pharmacy compounding.

Off-label use of medicines is widespread in rare diseases (Dooms et al., 2018), and while it is a useful way to serve unmet needs and drug shortages, it entails risks and uncertainties for patients and prescribers. Similarly, hospital exemptions and pharmacy compounding of approved OMPs serve the crucial purpose of meeting the needs of specific patients that cannot be met through approved and available OMPs (Dooms and Carvalho 2018).

However, when the off-label use of medicines and pharmacy compounding or hospital exemptions in the presence of an approved OMP go beyond serving the needs of individual patients, they create uncertainty for OMP developers around the validity of their market exclusivity or whether a large part of the market might be served by these medicines. In addition, they entail risks and uncertainties for patients and prescribers in relation to safety and efficacy.

To increase legal certainty and establish the validity of the 10-years ME incentive, the EMA and other national regulatory bodies should adopt EU-wide Good off label use guidelines and Guidelines clarifying the role of hospital exemption and pharmacy compounding. This will support healthcare practitioners in ensuring safe drug therapy when licensed medicines do not meet the needs of the individual patient, while making sure that public health remains a priority and is not undermined by solely cost containment considerations. Stakeholders have already identified a set of principles promoting good practices for the off-label use of medicines which should be used as a starting point for such guidelines by the EMA and other national regulatory bodies (Dooms et al., 2018).

#### Proposal 10. Adapt the Regulatory Pathway to the Specificities of OMP Groups With Additional Challenges

Given the heterogeneity of rare diseases and the OMP landscape, the regulatory pathway for OMPs can benefit from flexibility to accommodate for the specific challenges faced by certain groups of OMP development projects, two examples of which are OMPs indicated for extremely rare diseases and OMPs with multiple indications.

**Example 1**: OMPs indicated for extremely rare diseases could benefit from a tailored regulatory pathway. This is because the (even) smaller patient populations impose additional hurdles across the development path for these OMPs. In particular, conducting clinical trials and collecting sufficient evidence on safety and efficacy is more challenging with extremely rare diseases due to very small patient populations, imposing high risk and increased time to market for these OMPs. A way to adapt the regulatory pathway to the unique challenges of these OMPs would be to recognise extremely rare diseases as a part of a bigger group of similar diseases, building and expanding on the PRIME scheme and disease grouping done by e.g., the Rare disease Research Hub and ERNs. Essentially, this means that the EMA would accept a wider (yet still very applicable) scope of evidence in assessing safety and efficacy, and thereby reduce the hurdles of extremely small patient populations.

**Example 2**: The registration of multi-indication

OMPs could benefit from additional flexibility. Currently, the regulatory pathway does not take full advantage of the fact that a single active pharmaceutical ingredient can have the potential to treat multiple conditions. Differently from non-OMPs, OMP developers cannot freely extend an existing marketing authorisation to include a new indication. Each orphan indication can, under the current rules, only be included in the original marketing authorisation when it has an orphan designation and that designation is maintained at the time of approval of the new indication. This creates significant uncertainty for OMP developers and hinders the development of new (orphan) indications. It also implies a risk that when for a second indication the orphan designation is not maintained at the time of approval, the developer has to waive the orphan status of the initial indication so as not to delay the approval of the new indication. This undermines the objectives of the OMP Regulation.

The historical reason for this rule was to avoid confusion about the scope of the market exclusivities. This rationale has however disappeared as the Commission now operates a detailed public Union Register of centrally approved medicines, which provides full transparency on market exclusivity rights. Therefore, there can be no drawback to allowing for one marketing authorisation to contain orphan and non-orphan indications.

Based on these two examples, EU policy makers should investigate the need for and implement additional regulatory flexibility for specific groups of OMPs. While a flexible pathway decreases the burden in OMP development it may also increase complexity for regulators, ultimately leading to a more cumbersome system. Therefore, policy makers have the challenging task of striking a balance between flexibility and complexity.

### Need 4. Improving the Coherence and Predictability of Demand and Pricing for OMPs

Demand in the pharmaceutical sector involves many actors: patients have needs to be met, prescribers (mostly) choose the treatment plan for their patients, payers (i.e., health insurance companies, national healthcare systems) pay for the treatments that patients receive but also decide which treatments are available in their Member State and at which conditions.

**FIGURE 10:**
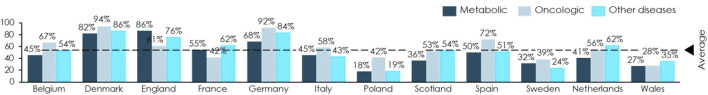
Share of reimbursed OMPs in selected EU Member States, by type of disease. Note: Between January 1995 and May 2000. Source: Copenhagen Economics based on historical average success rates from EMA data (European Medicines Agency 2020). Download medicine data. https://www.ema.europa.eu/en/medicines/download-medicine-data [Accessed april 21, 2021]).

After obtaining central marketing authorisation, OMP developers need to seek market access in each Member State where they intend to market their medicine. Based on the Member State’s specific procedures and requirements, each HTA body assesses the evidence available on efficacy of the OMP and forms an opinion on its relative value. The HTA assessment is then used to determine the level of reimbursement and is one of the core elements used by payers in price negotiations with OMP developers. The heterogeneous national process and procedures contribute to heterogeneous access to OMPs across EU Member States, see Figure 10.

Market access conditions are crucial incentives for the development of OMPs as they determine the level of revenue that each OMP will generate. Neglecting the complex and critical role of demand-side conditions in the OMP incentive framework will lead to suboptimal outcomes. This is because uncertainty concerning demand, the final price level and the size of the accessible market are crucial factors in the investment case for OMP development. Currently, the OMP Regulation provides supply-side incentives, such as protocol assistance and administrative and procedural guidance for SMEs, which are important elements in the overall OMP incentive framework. However**,** their potential can be maximised if aligned with the incentives on the demand side.

Today, market access in the EU Is characterised by two challenges in relation to development incentives.

First**,** the lack of alignment between payers, prescribers and patients’ needs creates uncertainty on the willingness to pay for OMPs. This uncertainty increases the perceived risk, thereby worsening the investment case for OMP development. This problem is especially pronounced in the case of innovative treatments with high prices. This is because payers’ willingness to pay is confronted with finite health care budgets put under strain by the growing number of innovative and high-price medicines. In addition, OMP developers often face challenges with having the value of their innovative treatments recognised by payers, despite having obtained a marketing authorisation. This is because the framework for value assessment is not suited to cater for the level/type of evidence of efficacy that the OMP environment allows to collect.

Second, the lack of alignment on the framework for conducting HTA assessments across Member States creates uncertainty on the size of the population that OMP developers will be able to access, on the access conditions and on the price levels achievable in different Member States. Moreover, the separate and different procedures create duplication of efforts and additional costs for both OMP developers and society at large.

To mitigate these challenges, policy makers at EU and national levels need to see market access as a crucial element in the OMP incentive framework and to seek ways to align demand-side incentives and procedures with the OMP development pathway. Improving the coherence and predictability of demand and pricing for OMPs will create an environment where incentives carry through the development path and where additional uncertainties for OMP development coming from the demand side are eliminated.

Next to these proposals, more wide-spread use of outcome-based pricing models in combination with a coherent RWE framework would further contribute to reducing uncertainties in pricing and reimbursement (P&R) negotiations, see Supplementary Box 4.

The OD Expert Group makes four policy proposals for improving the coherence and predictability of demand and pricing for OMPs, with present-day challenges in mind.

#### Proposal 11. Establish an Iterative Early Dialogue for EMA-HTA Bodies and OMP Developers

Currently, OMP developers have very little interaction with HTA bodies pre-authorisation. There exists no widely used formal process where OMP developers can discuss the clinical development of OMPs with HTA bodies and EMA. Early, more frequent and more efficient collaboration between OMP developers and HTA bodies would reduce uncertainty and increase efficiency of the regulatory process, market access and OMP development at large. For instance, early alignment on the evidence requirements for the value assessment of a specific OMP would reduce the uncertainty on whether the evidence produced at the development stage will also allow an effective value assessment at the market access stage.

In practice, this would mean establishing a framework where delegates from HTA bodies accompany OMP developers throughout the regulatory process, together with the EMA (as proposed under proposal 7). Building on the joint EMA-EUnetHTA (European Network for HTA) Scientific Advice framework^10^, this earlier involvement of HTA bodies would provide much needed early guidance on the type and amount of evidence required to assess the value of treatments with a high level of certainty. More seamless coordination between HTA bodies and OMP developers ultimately means that OMPs will reach the market quicker and will be accessible to a larger share of EU patients.

#### Proposal 12. Create a Common EU Value Assessment for OMPs

Today, requirements and assessment frameworks of HTA bodies diverge (at times considerably) across Member States, making market access an uncertain process with multiple, overlapping assessments. Harmonising the way in which HTA assessments are conducted will improve both patients’ access to treatment and certainty of market outcomes for OMP developers. This can be achieved by ensuring effective transnational cooperation in the form of a common EU framework for value assessment or ideally, an EU-wide HTA process for OMPs.

The European Commission proposal for an EU HTA regulation currently discussed by the Parliament and the Council could play a role in this recommendation, provided the adopted text ensures a sufficient level of flexibility to manage evidential uncertainty in specific cases, such as for OMPs. Managing evidential uncertainly means, inter alia**,** that the guidance developed for the joint clinical assessment of OMPs under the EU HTA Regulation should be “progressive” i.e., inclusive of sources of evidence beyond randomised clinical trials. On this point we refer to our proposal 4 on establishing a coherent policy framework for the use of RWE.

A common value assessment framework, building on the EU HTA Regulation, would explicitly define how clinical value is determined, what evidence is required and how evidence is used in the value assessment. It will also have to build upon and inform the early dialogue between HTAs and OMP developers (see policy proposal 11). This process should be aligned with the previous stages of the regulatory pathway, such that evidence requirements and evidence assessments are consistent.

In particular, the EU value assessment should incorporate the European Commission’s decision on assessment of Significant Benefit at the time of marketing authorisation.

Importantly, a future common EU value assessment framework for OMPs should be designed to fit the specificities of rare diseases. This is currently not the case in most EU member states. On the contrary, the traditional cost-effectiveness (CE) assessments that are usually applied to OMPs systematically generate unfavourable outcomes for rare conditions. This is because traditional CE frameworks focus on incremental CE ratios, often expressed as cost per quality-adjusted life year gained as a measure of cost per patient. By definition, this ratio cancels out the size of the numerator and the denominator, and hence any differences grounded in the prevalence or rarity of a disorder. However, evidence shows that citizens place value on living in a society that does not leave behind its weakest members, such patients suffering from rare diseases (Schlander et al., 2014; Richardson and Schlander 2019). Such a social preference may be captured by measures of the “social willingness-to-pay” of citizens for the availability of ODs to patients in need. This makes the case for reconsidering traditional value frameworks for ODs and for giving more prominence to the (limited) budget impact of ODs as opposed to the cost per patient in cost value analyses (Schlander et al., 2018).

A common EU value assessment could be established through the existing EUnetHTA, which already supports HTA cooperation within the EU. An EU-wide HTA process would take this a step forward by not only building a common framework and cooperation but actually conducting one unique assessment recognised across Member States.

A joint assessment of the value of OMPs will be a crucial prerequisite for a common access pathway (see policy proposal 13). In fact, the proposed common access pathway would not be feasible without a joint assessment that is binding on all participating Member States and forms the basis of discussions on pricing. It is important to note, however, that a common EU value assessment, which provides the basis for P&R negotiations, comes with clear challenges: there are still significant differences between national health systems in terms of clinical practice, patterns of medicine usage, as well as affordability. Therefore, when deciding on the suitability of joint efforts, legal, political and economic challenges need to be taken into account when choosing the most appropriate tools to foster access to medicines.

In the future: link the need for strong demand-side incentives with the EU’s goal to foster wider and more equal access to OMPs.

The OD Expert Group did not set out to develop proposals on the goal of wider and more equal access for patients to OMPs across the EU. Nevertheless, OMP development incentives on the demand-side and the breadth of market access are linked. For instance, centralised market access procedures at the EU level can mean more predictability of demand and larger markets for OMP developers while also ensuring more equal access conditions for patients.

While centralised market access for OMPs may not be possible under the current distribution of EU competences and its crucial pre-conditions (e.g., a common EU value assessment) are not yet in place, the OD Expert Group urges policy makers to already now study its feasibility and, where possible, test it in pilots. Therefore, the OD Expert Group makes two further proposals.

In this context, it is however important to note that access inequalities will not be solved solely by changes to the OMP incentive framework. In parallel, many issues with and barriers to access need addressing, taking into account specific national policies and circumstances (EFPIA 2020).

#### Proposal 13. Pilot a Common EU Access Pathway for “Priority” (e.g., Extremely Rare) OMPs

Decentralised and de-harmonised pricing negotiations, as they currently exist in Europe, do not only increase uncertainty for OMP developers, but they also affect patient access. A common EU access pathway for OMPs across Europe would be a transformative step in strengthening payers’ ability to reap value from improved OMP incentives and to simplify and equalise access conditions. Such a common EU access pathway, comprising of joint price negotiations, could be applicable for OMPs addressing extremely rare diseases—for which access conditions are even more difficult.

Any joint price negotiations by Member States or led by the European Commission must build on a joint assessment of the value of the product, which is binding to all participating Member States, and needs to be the basis of any pricing discussions. Moreover, any joint negotiation effort has to take account of the unique legal, political and economic challenges it brings about owing to the differences between national health systems in terms of policy goals, clinical practice, patterns of medicine usage, as well as medicine pricing and reimbursement.

Considering all caveats and preconditions, a common EU negotiation alliance could be a useful forum to develop ways to overcome the challenges that market access poses to very specific groups of OMPs. For instance, common negotiation could be tested as a pilot in the context of specific extremely rare diseases, where EU Member States could procure medicines based on a common fund that aims at achieving market access for all known patients across the EU.

#### Proposal 14. Facilitate Homogeneous Access to OMPs Across EU Member States

A further way to grant more equal access for patients across the EU could be to create an incentive-based Special Access Program for OMPs. OMP developers would have the opportunity to sign up to the program which would require them to market their OMP in a selected number of countries in return for defined rewards. These rewards could for instance be an additional year of exclusivity, either as an addition to OMP market exclusivity or as an extension of the supplementary protection certificate, 5 years after market access in the first Member State.

The Special Access Program would operate under minimum transaction costs with fixed low OMP prices for eligible countries to be defined by the European Commission.

Prior to implementing any such program, a thorough impact assessment must be carried.

Out, which also acknowledges potential unintended consequences. For instance, countries’ use of external reference pricing and these consequences could be a result of non-eligible so-called parallel imports exploiting the opportunities of the Single Market.

The Special Access Program would introduce a radically different commitment by all stakeholders to work for more equal access across the EU. The programme can only be successful if designed in union between the EU, industry and potentially eligible Member States.

## Conclusion

The 14 policy proposals are a further step towards achieving the goal that EU policy makers set for themselves 20 years ago: achieve the same quality of treatment for rare disease patients as other patients within the European Union. Today, the proposals also align with the policy ambitions of an improved R&D ecosystem and new incentive models for OMPs that the European Commission has set out in the EU Pharmaceutical Strategy.

Such a commitment should take the form of a Commission communication accompanying the OMP Regulation, which outlines the ambitions and policy action the EU pursues to improve the OMP development framework in Europe. Only an ambitious policy agenda can bring about the quantum leap needed to address unmet needs of rare disease patients today.

The OD Expert Group calls upon EU policy makers to endorse and commit to a wider, ambitious policy agenda for OMP development that includes the remainder of the proposals. The OD Expert Group is aware that additional topics will need to be discussed, such as in depth discussions on the level of real world evidence needed, the ethical implications of our proposals with regards e.g., to intellectual property rights and extended market exclusivity, and the involvement of private partners or venture capital firms in drug development. These are beyond the scope of this work, but we hope the work will form a basis to initiate these further discussions.

## Consortium Economics A/S

The Copenhagen Economics members who were involved with this work are Laura Virtanen and Julia Sabine Wahl.

## The OD Expert Group

The OD Expert Group is a cross-disciplinary group of experts representing different stakeholders in the rare disease community. The group includes experts from research, academia, patient groups, rare disease companies, investors and trade associations (see https://od-expertgroup.eu/members/ for a full list of members).

## Footnotes

1 European Medicines Agency (2021a). Orphan incentives. https://www.ema.europa.eu/en/human-regulatory/research-development/orphan-designation/orphan-incentives [Accessed april 26, 2021]

2 Note that there may be treatments available for some of the 95% of rare diseases without an authorised OMP such as off-label prescriptions (see e.g., https://www.medicinesforchildren.org.uk/unlicensed-medicines)

3 According to GlobalData Pharma Intelligence data, between the years 2016–2019, there were 1,039 unique OMPs in the development pipeline (in pre-clinical, clinical IND/CTA, and pre-registration stages) in the US compared to only 483 in Europe

4 Markets Insider (2021). Global Cell and Gene Therapy Market to Reach $11.96 Billion by 2025. https://markets.businessinsider.com/news/stocks/global-cell-and-gene-therapy-market-to-reach-11-96-billion-by-2025-1028421352 [Accessed april 20, 2021]

5 Gainottiet al.(2018). The RD-Connect Registry & Biobank Finder: a tool for sharing aggregated data and metadata among rare disease researchers. https://pubmed.ncbi.nlm.nih.gov/29396563/[Accessed August 4, 2021]

6 EJPRD (2021). European Joint Programme Rare Diseases. Coordinated Access to Data and Services. https://www.ejprarediseases.org/coordinated-access-data-services/ [Accessed april 20, 2021]

7 European Commission (2021). European Platform on Rare disease Registration (EU RD Platform). https://eu-rd-platform.jrc.ec.europa.eu/_en [Accessed april 20, 2021]

8
https://www.ema.europa.eu/en/human-regulatory/research-development/prime-priority-medicines

10 European Medicines Agency 2021c). Parallel consultation with regulators and health technology assessment bodies. https://www.ema.europa.eu/en/human-regulatory/research-development/scientific-advice-protocol-assistance/parallel-consultation-regulators-health-technology-assessment-bodies [Accessed april 20, 2021]

## Glossary

AI: Artificial intelligence
CE: Cost-effectiveness
CHMP: Committee for Medicinal Products for human use
COMP: Committee for Orphan Medicinal products
EC: European Commission
EFPIA: European Federation of Pharmaceutical Industries and Associations
EIB: European Investment Bank
EJP RD: European Joint Programme on Rare Diseases
EMA: European Medicines Agency
ERN: European Reference Network
EU: European Union
EUCOPE: The European Confederation of Pharmaceutical Entrepreneurs
EUnetHTA: European Network for Health Technology Assessment
FAIR: Findable, accessible, interoperable and reusable
HTA: Health Technology Assessment
IRDiRC: International Rare Diseases Research Consortium
MA: Marketing Authorisation
NBS OD: Newborn screening Orphan Drug
ODD: Orphan Drug Designation
OMP: Orphan medicinal product
ODTC: Orphan Drug Tax Credit
PPP: Private-Public Partnership
P&R: Pricing and reimbursement
RWE: Real-world evidence
R&D: Research and development
SME: Small and medium-sized enterprise
VC: Venture Capital
WES: Whole-exome sequencing
WGS: Whole genome sequencing
