# Supplementary material for: Orphan Medicine Incentives: How to Address the Unmet Needs of Rare Disease Patients by Optimizing the European Orphan Medicinal Product Landscape Guiding Principles and Policy Proposals by the European Expert Group for Orphan Drug Incentives (OD Expert Group)
Source: Front Pharmacol. 2021 Dec 16;12:744532. doi: 10.3389/fphar.2021.744532 (PMC8717920; doi:10.3389/fphar.2021.744532)
Supplement: Supplementary file 1 [file DataSheet1.docx]

**Supplementary Table 1: Glossary of key terminology**

| **Attrition rate** | Percentage of drug development projects abandoned at different stages of development or market access |
| --- | --- |
| **Incentive** | Any measure meant to promote the development of medicines to treat rare diseases |
| **Indication** | The labelled use of a specific drug (an OMP) for treating a particular disease |
| **Investment case** | Assessment of the viability of an investment from an investor’s perspective |
| **Marketing Authorisation (MA)** | The approval to market a medicine in European Union Member States |
| **Market required return on investment** | Minimum level of return required for an investment given the level of risk present |
| **Market Exclusivity** | Period after the marketing authorisation of an orphan medicine when similar medicines for the same indication cannot be placed on the market |
| **Return on investment (ROI)** | A measure for the amount of return on a particular investment, relative to the investment’s cost. *Ex-ante ROI:* estimated return that investors can expect to earn from an investment at the end of a specific period. *Expected ROI:* the anticipated profit or loss on an investment that takes into consideration systematic and unsystematic risk |
| **Orphan Drug Designation (ODD)** | A status assigned to a medicine intended for use against a rare condition. The medicine must fulfil certain criteria for designation as an orphan medicine so that it can benefit from specific incentives |
| **Outcome-based pricing** | Pricing of a product (OMP) based on perceived outcomes (e.g. value to patients and to society at large), and not costs |
| **Real-world evidence** | Evidence on the usage and potential benefits or risks of a medical product derived from analysis of (real-world) data |
| **Supplementary protection certificate (SPC)** | An intellectual property right that serve as an extension to a patent right |

**Box 1: The investment perspective**

Investors, in this case **OMP developers**, commit resources at the onset of an OMP development project. They take the decision to invest in OMP development based on their expected return given the expected costs, risks, and timeline of the development project and the expected revenues.

**Costs:** OMP development is indisputably costly. Significant investments are required all along the development path, from pre-clinical and clinical trials, to regulatory approval and securing market access, to production and post-market access activities. All else equal, the higher the expected costs are for bringing a medicine to the market, the higher the expected return will need to be to make the investment worthwhile.

**Risks:** Investments in OMP development are pursued only if investors expect to break even and earn a return commensurate with the **risk.** Bringing an OMP to the market entails significant risks**,** such as the risk of failure along the OMP development path or the regulatory risk of losing orphan designation.^a^ In addition, not all medicinal products that reach the market are successful in generating revenues. The risk is reflected in the difference between expected returns (determined from future prices and patient demand) and required returns (as expected at the time of the investment).

All else equal, the higher the perceived risks in bringing a medicine to the market, the higher the expected return needed to make the investment worthwhile.

**Time to market:** The time needed from the start of the project to patient delivery also affects the investment case. The longer the timeline, the higher the expected return needed to make the investment worthwhile. The timeline is affected by multiple factors, such as the level of relevant knowledge already available and the speed of proceeding through the regulatory pathway.

**Expected revenues:** The expected revenue, i.e. the size of the patient population times the expected price, determine whether an investment is worthwhile given expected costs and risks. The patient population depends on the disease prevalence, the product’s therapeutic characteristics, and the success of market access procedures in different countries and prescribing practices.

The price is determined through negotiations at the Member State level, which take into account a multitude of elements (level of available evidence, patient value, comparator prices, budget impact).

Incentives, i.e. policy measures meant to promote the development of medicines to treat rare diseases, can act on all of the above elements: by lowering costs, reducing risk or making it more manageable, shortening the time needed to go through the development path or by increasing/securing the return.

Note: a) Approximately only one in ten candidate compounds that enter the clinical trial phase will succeed in obtaining regulatory approval and generate (some level of) revenues. See for instance, Alacrita (2018).


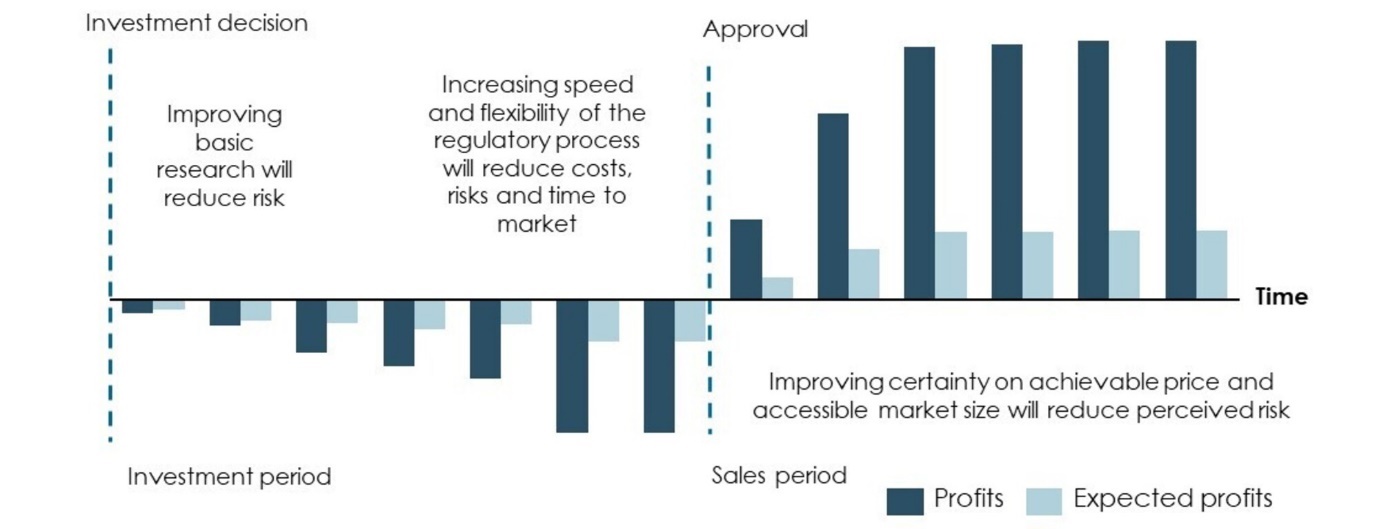


**Figure Box 1: Tackling the identified challenges improves the OMP investment case (illustrative)** Note: Illustrated example.

Source: Copenhagen Economics and the OD Expert Group.

## Box 2 Considerations for a modulated approach to OMP incentives

Some of the proposals presented in the previous section pursue a more modulated approach to OMP incentives. While the case for modulated incentives is identifying appropriate solutions at various levels, ways to apply such modulated incentives is still a challenging task. When designing a modulation mechanism, policy makers should therefore respect four important considerations:

**1. Pursue a holistic framework for unmet needs**

Modulation is a tool to capture the heterogeneity of investment cases across OMP development projects and allows to direct specific incentives into certain rare disease areas. While the regulatory framework may adopt a modulated approach across different OMPs, the concepts of unmet need and orphan designation should continue to apply broadly and not by themselves serve as tools for modulation. This is because the definition of these concepts has impacts that go far beyond the setting and designing of OMP incentives.


Today, there is no agreed common definition for the concept of unmet medical need and the concept varies in content and in meaning for different stakeholders (e.g.  patients, developers, clinicians, regulators, HTA authorities, payers) and over time. Unmet needs do not only exist where there is no authorised treatment for rare diseases, but depending on disease severity, burden of the illness and impact on patient quality of life, the absence of transformative and curative treatments also qualifies as an unmet need. Moreover, the indirect burdens for families and caregivers are essential elements of unmet need. The definition of unmet need has impacts all along the OMP pathway, but in particular on the perceived value of the treatment. On top of that, the concept of unmet need has important overlaps with other regulatory concepts, such as significant benefit.

Against that background, a legally binding, restricted definition of unmet need that guides the modulation of incentives, i.e. by limiting (additional) incentives to a strictly defined area of unmet need, is not an appropriate policy tool. Instead, a broad, holistic unmet need framework can recognise the many ways in which unmet needs manifests itself all while attracting developers into underserved rare disease areas. Multi-stakeholder dialogue along the OMP development path, including patient representatives, developers, clinicians, regulators, HTA experts and payers, can then allow to continuously refine and update existing assumptions on unmet needs.

Today, the ODD allows OMP developers to attract investment already at the very early stages of development. This is crucial since OMP developers need to make investment decisions many years before a product reaches the market. The current ODD based on a 5 in 10,000 prevalence threshold ensures that developers can make early-stage decisions. It is therefore prudent to maintain it as the main criterion for the ODD award.

**2. Understand the heterogeneity of investment cases and underlying drivers**

Any policy which modulates incentives must rely on a solid understanding of the variety of reasons behind the lack of investment in various groups of diseases. Investigating which groups of diseases suffer from, e.g., lack of basic research, from the infeasibility of conducting clinical trials, or from countries’ low willingness to pay is key and the first step to modulating incentives effectively. This requires policy makers to conduct a separate, thorough study of i) areas where current incentives may be too weak (where authorised treatment is currently lacking) and ii) areas where incentives already appear to be strong (“crowded areas”). Such a study should closely involve experts in rare disease development.

**3. Design an appropriate selection mechanism**

A modulated approach to OMP incentives requires a selection mechanism, which differentiates OMPs according to their unique investment case and allows for modulating incentives accordingly. Such a mechanism should allow for incentives to be aligned with the challenges that different groups of OMPs face along the development path. Establishing such a selection mechanism is not a straightforward task, as the investment case may not be simple to assess and can change over time.

Moreover, such a mechanism should avoid both type I errors (granting additional incentives for development projects that do not actually need them) and type II errors (failing to incentivise development projects that do require additional incentives).

Additionally, EU policy makers should consider several ways of selecting groups of “priority diseases” based on the key characteristics that drive a weak investment case for OMPs and where the unmet need is deemed greatest. Two examples are:

1. By disease rarity through defining thresholds of eligibility for additional incentives: this can be an additional prevalence criterion for identifying extremely rare diseases that are systematically underserved. For instance, this could include diseases that affect less than 1 in 50,000 people.
2. By disease areas through a top-down definition of target areas where more development should take place**:** this could be based on pre-defined groups of rare diseases which require additional attention, such as paediatric rare diseases, or those for which there are no authorised treatments or for which there is a lack of research.

Any selection mechanism will inevitably come with caveats and pitfalls. For instance, a categorisation of diseases based on thresholds and other criteria will necessarily mean that drug developers and regulators will dedicate many resources to determining whether certain development projects should be eligible. Since a definition of categories can never be perfect, type I and II errors may occur.

**4. Set equal incentives at the margin**

To target areas without authorised treatment, incentives should be designed such that, on the margin, the investment case for developing these medicines for priority diseases is as attractive as for any other type of (orphan) medicinal product. A modulated approach which sets incentives according to the investment case of different categories of OMPs may naturally allow for a balancing of incentives, whereby incentives are reduced from one group to fund the additional incentives for the other. This would imply a combination of upward and downward modulation**.**

While modulation has the potential to truly improve the situation for patients suffering from rare diseases that currently lack treatment, it can also have adverse effects on innovation and regulatory efficiency, which may leave other groups of patients worse off. The need for modulation should therefore be balanced with the need for transparency and efficiency of the system, and any modulation of OMP incentives needs to be preceded by an impact assessment.

**Box 3: The EC decision on Significant Benefit should be binding for Member States**

According to **Article 3(1) of Regulation (EC) 141/2000** an ODD is only granted if there is *“no satisfactory method of diagnosis, prevention or treatment of the condition in question that has been authorised in the Community or, if such method exists, that the medicinal product will be of significant benefit to those affected by that condition.”* (European Commission 1999, 3). Significant benefit is defined in **Article 3(2) of Regulation (EC) No 847/2000** as *“a clinically relevant advantage or a major contribution to patient care”* (European Commission 2000, 3). In other words, an ODD is only granted by the European Commission if there is no therapy alternative or if the OMP in question is better than the existing therapy alternatives.

There is no doubt that the EU Member States have the competence to regulate the prices of medicines. However, Member States and their authorities have to comply with EU law when exercising their power to regulate the price of pharmaceuticals*.* In addition, the EU courts have underlined that a broad interpretation of the concept of OMP market exclusivity is needed to ensure its effectiveness and that off-label prescribing *“should not be facilitated****”*** (European Union 2017, 141) in the presence of a similar approved OMP***.*** The General Court of the EU has recently also highlighted that in decisions concerning the maintenance of an ODD, an off-label use comparator should not be considered. The inception impact assessment on the OMP Regulation states that *‘’account should be taken of the jurisprudence of the EU courts with regard to the designation criteria for orphan medicinal products’’* (European Commission 2020b, 5). The aforementioned rulings are important milestones in providing stability and in confirming the value of an ODD.

Lastly, **Article 288 (4)** of the Treaty on the Functioning of the EU explicitly states that a *“decision shall be binding in its entirety”* (European Union 2012, 172)*.* Therefore, the EC’s decision on Significant Benefit should be taken into account in national pricing and reimbursement decisions.

**Box 4: Outcome-based pricing models as a tool to reduce uncertainty for innovative OMPs**

Innovative and personalised treatments have high prices due to high development costs and risks and the small patient population which they serve. While these treatments offer great value to patients, their high prices challenge national healthcare budgets and compromise patient access. In addition, due to the limited clinical evidence, it may be difficult for many OMPs to demonstrate a satisfactory level of value that is recognised by payers. Outcome-based pricing looks beyond the upfront cost of OMPs, as perceived by payers, by assigning a monetary value to the achieved patient, societal and budget outcomes, see Figure.

Outcome-based pricing models could lessen the perceived risk associated with reimbursement, and particularly conditional reimbursement, of expensive treatments – by holding OMP developers accountable for the delivery of value to patients. In other words, outcome-based models ensure that any increase in price is aligned with a proportional increase in value. This requires that patient outcomes of OMPs are studied and reported through high-quality RWE, but also that RWE is accepted as a standard form of evidence in reimbursement market access procedures. Outcome-based pricing models are a useful tool for market access; their use is by no means limited to a common EU negotiation alliance.


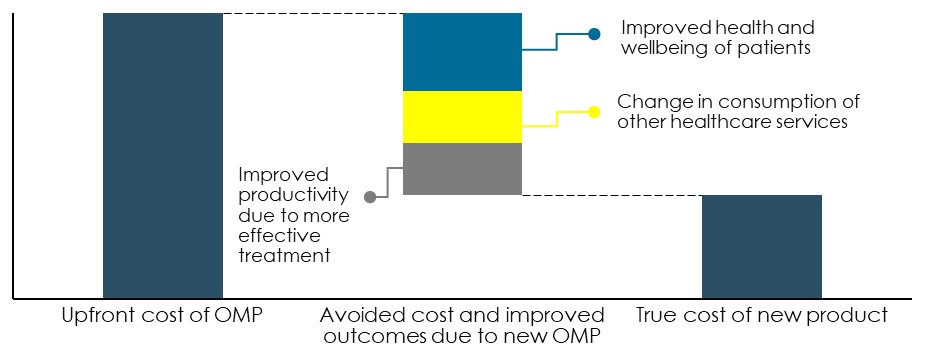


**Figure Box 4: Illustrative example of calculating the true cost of an OMP for payers**

Note: Illustrated example.

Source: Copenhagen Economics and the OD Expert Group.
